# Harmonic distortion reduction and dynamic stability in PMSG-CHBI wind energy systems via a dual optimization–prediction approach

**DOI:** 10.1038/s41598-026-35707-y

**Published:** 2026-01-26

**Authors:** Lijo Jacob Varghese, G. Venkatesan, Aymen Flah, Monia Hamdi

**Affiliations:** 1 Department of Electrical and Electronics Engineering, Sri Krishna College of Engineering and Technology, Coimbatore, Tamil Nadu India; 2 Department of Electrical and Electronics Engineering, CARE College of Engineering, Tiruchirappalli, 620009 Tamil Nadu India; 3 Applied Science Research Center, Applied Science Private University, Amman, 11931 Jordan; 4 ENET Centre CEET VSB-Technical University of Ostrava, Ostrava, 708 00 Czech Republic; 5 Centre for Research Impact and Outcome, Chitkara University, Punjab University, Punjab, Rajpura, 140401, India; 6 Department of Information Technology College of Computer and Information Sciences, Princess Nourah bint Abdulrahman University, P.O. Box 84428, Riyadh, 11671 Saudi Arabia

**Keywords:** Cascaded H-bridge inverter (CHBI), Permanent magnet synchronous generator (PMSG), Total harmonic distortion (THD), Power quality, Optimization, Greater cane rat algorithm (GCRA), Visual Relational Spatio-Temporal Neural Network (VRSTNN), Energy science and technology, Engineering

## Abstract

Permanent Magnet Synchronous Generators (PMSGs) combined with Cascaded H-Bridge Inverters (CHBIs) are widely adopted in wind energy systems due to their high efficiency and superior power quality. However, even five-level CHBIs retain noticeable low-order harmonic components and output ripple under nonlinear PMSG wind conditions, indicating that further refinement of switching-angle control is required to maximize performance. This paper introduces a dual optimization–prediction framework to address these challenges. The proposed method integrates the Greater Cane Rat Algorithm (GCRA) for adaptive switching-angle optimization with a Visual Relational Spatio-Temporal Neural Network (VRSTNN) for predictive control under dynamic operating conditions. By jointly minimizing harmonic distortion and forecasting system responses under varying wind and load scenarios, the framework ensures high-quality voltage output and stable operation. Across 10 independent simulation runs, the system achieved a mean THD of 2.10% ± 0.04, voltage ripple of 1.6% ± 0.12, and response time improvement from 0.035 s to 0.012 s, confirming consistent performance with low variability. MATLAB results further demonstrate reduced power losses, improved efficiency, and faster transient stabilization compared with ANN, RERNN-LSE, RPOA-DTRN, GA–PSO, and CNN-based methods. These findings highlight the potential of the dual optimization–prediction strategy as a robust and scalable solution for next-generation intelligent PMSG–CHBI wind energy conversion systems.

## Introduction

Wind energy has emerged as a vital renewable resource due to its sustainability and its potential for seamless integration into modern electricity systems^1^. Reliable operation of wind turbine (WT) systems requires maintaining voltage stability, minimizing energy losses, and ensuring acceptable limits of power quality^2–4^. Harmonic distortion, voltage ripple, and poor dynamic response are among the most pressing challenges, as they directly affect the efficiency, reliability, and lifespan of power electronic components^5–8^. Improving harmonic suppression, conversion efficiency, and transient stability has therefore become central to advancing the performance and long-term reliability of wind energy generation systems^9,10^.

Despite ongoing progress, wind energy generation systems continue to face operational challenges. Variable wind speeds introduce irregular power output, complicating voltage stability and dynamic regulation. Multilevel inverters (MLIs), commonly employed in such systems, are prone to harmonic distortion, switching losses, and reduced waveform quality. Further issues, such as voltage ripple, frequency deviations, and thermal stresses, degrade system efficiency and grid compatibility over time. Overcoming these limitations requires advanced optimization and intelligent control strategies that can adapt to nonlinear dynamics under fluctuating operating conditions.

In recent years, several studies have investigated advanced control and optimization strategies for MLIs in renewable energy systems. Techniques based on artificial neural networks (ANNs)^11^, recurrent error-based neural networks (RERNN-LSE)^12^, hybrid metaheuristic–deep learning methods (RPOA-DTRN)^13^, and intelligent evolutionary approaches such as GA–PSO^14^ have shown improvements in harmonic reduction and control adaptability. Deep learning models, including convolutional neural networks (CNNs), have also been applied for nonlinear control and maximum power point tracking in variable wind conditions^15–17^. More recently, research has extended toward enhancing both power quality and energy efficiency of MLIs using intelligent algorithms^14^, while advanced control strategies have been developed to improve grid-tied inverter performance and ensure robust power quality^18^. Efforts have also focused on integrated microgrid management, where clustering and ANN-based models optimize scheduling and energy distribution for grid-connected hybrid systems^19^. Studies have demonstrated that combining hybrid renewable energy sources with MLIs and power quality conditioners significantly improves stability, harmonic suppression, and grid compliance^20^. Comprehensive reviews have further underscored the central role of MLIs in mitigating harmonics and supporting smart grids powered by renewable energy^21^. Additional contributions have investigated transactive energy management frameworks to improve scheduling and storage utilization in renewable-based microgrids^22^. Furthermore, predictive direct power control techniques integrated with PV-interfaced MLIs have shown promise for enhancing grid-connected power quality^23^. Other studies have explored multiple MGs with EV charging under a GJO-PCGAN framework, hybrid wind–pumped hydro–CAES–fuel cell configurations for techno-economic evaluation, and EV performance enhancement through buck–boost converter integration^24,25^. Additionally, graph- and neural-based methods have been proposed for PQ enhancement and voltage regulation in DC MGs and distribution systems, while reconfigurable PV–wind MGs have been optimized for dispatch strategies^26,27^. Collectively, these advancements demonstrate that hybrid optimization and intelligent control significantly enhance reliability, PQ, and sustainability in renewable-based power systems.

Recent studies have also emphasized the role of adaptive control strategies in enhancing power quality for multilevel inverter-based wind energy conversion systems. Advanced PWM and interleaving schemes have been proposed to suppress circulating currents, reduce harmonic distortions, and minimize common-mode voltage, thereby improving inverter efficiency and stability^28,29^. Transient stability analysis techniques and cooperative AC/DC voltage margin control approaches have been introduced to mitigate voltage violations in hybrid distribution networks^30,31^. In offshore applications, adaptive observer-based and funnel control strategies have been applied to ensure stable frequency–voltage support^32^, while stability estimation methods have been developed for DFIG-integrated wind farms^33^. On the economic energy management side, coordinated energy storage with hybrid demand response strategies has demonstrated significant potential in achieving cost-effective scheduling of wind-integrated microgrids^34,35^. Recent works have advanced metaheuristic-based inverter co-design^36^, predictive energy management for interconnected microgrids^37^, and hybrid optimization–machine-learning approaches for renewable energy forecasting^38^. Further studies have applied intelligent optimization techniques to enhance power quality in CHBI-based and hybrid renewable systems^39^, as well as improved multiobjective algorithms for grid-connected power-quality enhancement^40^. Although numerous studies have advanced harmonic mitigation and intelligent control for PMSG–CHBI systems, several critical limitations remain unresolved. Conventional ANN- and CNN-based controllers can learn nonlinear patterns but struggle to simultaneously manage (i) low-order harmonic interference produced by multilevel switching, (ii) rapid DC-link voltage fluctuations caused by variable wind and load conditions, and (iii) the multi-dimensional switching-angle search space inherent to CHBIs. Hybrid metaheuristic methods such as GA–PSO and RPOA–DTRN improve optimization but often converge slowly or stagnate in local optima, while many predictive and adaptive schemes impose high computational burdens that restrict real-time deployment. Importantly, most existing approaches treat harmonic suppression and dynamic voltage regulation as separate tasks, leading to suboptimal trade-offs between THD reduction, power loss, and transient response under nonlinear operating conditions.

To overcome these limitations, this work introduces two complementary techniques: the Greater Cane Rat Algorithm (GCRA) and the Visual Relational Spatio-Temporal Neural Network (VRSTNN). GCRA is inspired by hierarchical foraging and cooperative movement behavior in cane rat groups, enabling an effective balance between exploration and exploitation for high-dimensional switching-angle optimization. VRSTNN, although operating on numerical rather than visual data, is designed to capture relational dependencies and temporal evolution across system variables—analogous to visual relational reasoning—making it well suited for modeling nonlinear generator–inverter dynamics. By integrating GCRA’s global optimization capability with VRSTNN’s predictive spatio-temporal learning, the proposed framework aims to jointly suppress harmonics, stabilize DC-link dynamics, and enhance adaptive performance under uncertain wind conditions.

The main contributions of this study are summarized as follows:

- A novel GCRA–VRSTNN framework is developed for PMSG-based wind turbines with a three-phase, five-level cascaded H-bridge inverter (CHBI).
- The GCRA optimizes inverter switching angles and operational parameters, achieving reduced THD and improved conversion efficiency.
- The VRSTNN enables accurate modeling of nonlinear system dynamics for adaptive control under fluctuating wind speeds.
- The framework enhances voltage quality, minimizes power ripple and conversion losses, and ensures faster transient response compared to existing methods.
- Comparative analysis validates the superior robustness and performance of the proposed method against state-of-the-art techniques.

The novelty of this work lies in the combined use of GCRA and VRSTNN, offering a unified solution that simultaneously minimizes THD, reduces losses, and enhances dynamic stability. By outperforming existing optimization and neural-based approaches, the proposed framework provides a significant advancement toward efficient, reliable, and high-quality wind energy conversion.

The remainder of the paper is structured as follows. Section 2 describes the PMSG-based WT system and CHBI configuration. Section 3 outlines the proposed GCRA–VRSTNN method for minimizing THD and optimizing performance. Section 4 presents the results and discussion. Section 5 concludes the manuscript.

## Configuration of PMSG-based WT system and CHBI

**Fig. 1:**
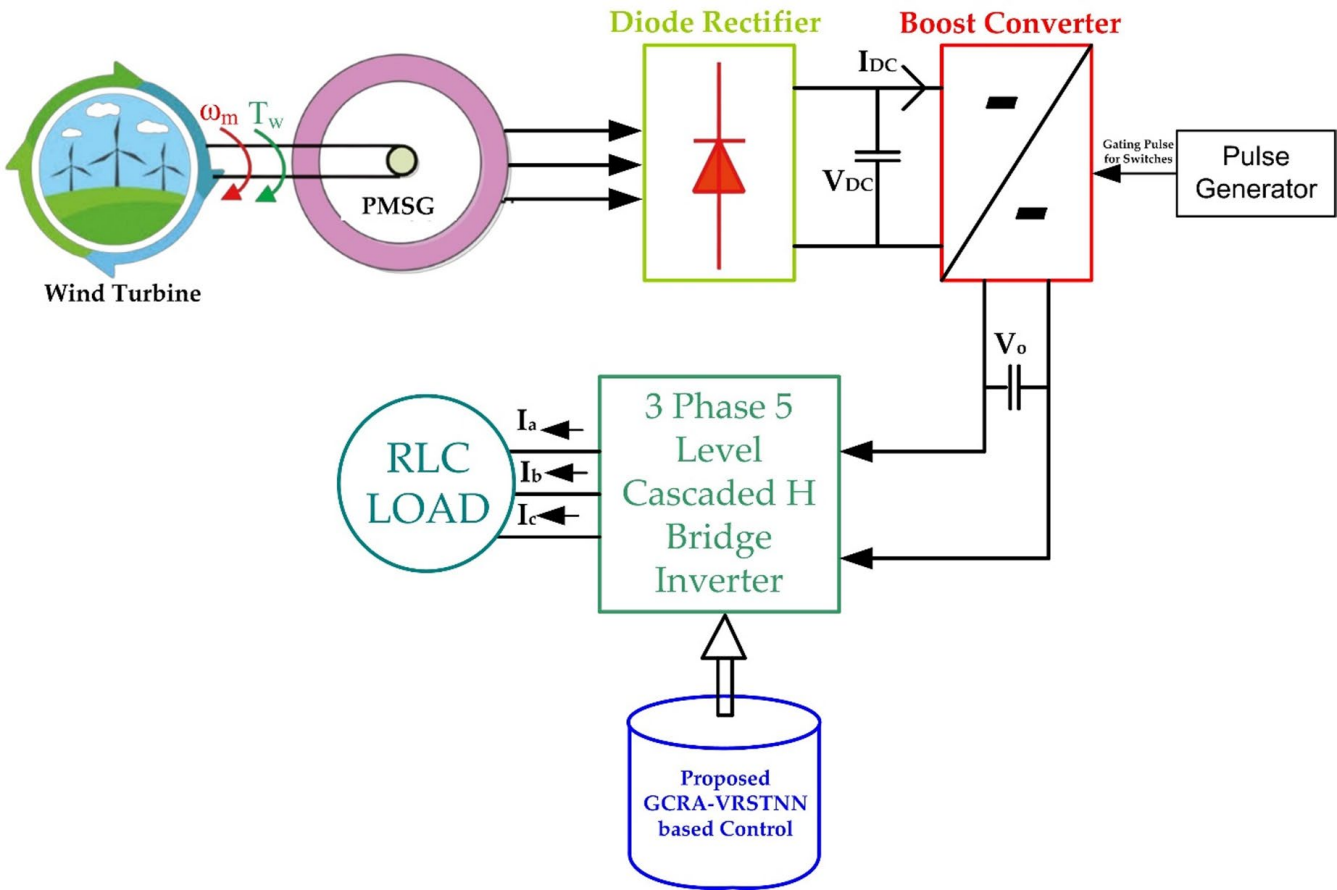
Structure of PMSG-based WT system and CHBI.

Figure 1 illustrates the structure of the suggested wind energy system. A PMSG is directly coupled to the WT to convert mechanical energy into electrical power. Generated three-phase AC power is fed into a 3 phase 5 level CHBI, which is responsible for converting and regulating the output into a suitable form for grid or load integration. The operation of the CHBI is controlled through two key components. First, the GCRA is applied to determine and optimize the switching angles of the inverter, ensuring efficient operation as well as reduction of unwanted harmonics. Second, VRSTNN is used to model and predict the dynamic behavior of the system, allowing adaptive control under varying wind as well as load conditions. Together, this structure integrates the PMSG, the CHBI, the GCRA, and the VRSTNN into a coordinated framework that represents the proposed intelligent optimization method.

### Different components of the dynamics of PMSG-based wind energy

WT with PMSG are used to capture the mechanical energy conversion of WECS, and PMSG is then extracted to WT. Here, mechanical energy is transformed into electrical energy. The WT shaft is directly attached and provides the rated PMSG torque, which produces voltage and current due to the gearbox’s support.

#### WT modelling

The aerodynamic power extracted by the wind turbine can be expressed as^41^:

1 $$\:\begin{array}{cccc}&\:{\mathrm{P}}_{\mathrm{W}\mathrm{T}}=\frac{1}{2}{\hspace{0.17em}}{\uprho\:}{\hspace{0.17em}}\mathrm{A}{\hspace{0.17em}}{\mathrm{C}}_{\mathrm{p}}({\uplambda\:},{\upbeta\:}){\hspace{0.17em}}{\mathrm{v}}_{\mathrm{w}}^{3}&\:&\:\end{array}$$

where

ρ = air density (kg/m³)

$\:\mathrm{A}={\uppi\:}{\mathrm{R}}^{2}=$ rotor swept area (m²),

$\:{\mathrm{C}}_{\mathrm{p}}=$ power coefficient (dimensionless),

$$\:{\mathrm{v}}_{\mathrm{w}}=$$ wind speed (m/s),

$\:{\uplambda\:}=\:$tip-speed ratio,

$$\:{\upbeta\:}=$$ pitch angle (°).

The power coefficient $\:{\mathrm{C}}_{\mathrm{p}}$is a nonlinear function of $\:{\uplambda\:}$and $\:{\upbeta\:}$and is typically approximated as:

2 $$\:\begin{array}{cccc}&\:{\mathrm{C}}_{\mathrm{p}}({\uplambda\:},{\upbeta\:})={\mathrm{C}}_{1}(\frac{{\mathrm{C}}_{2}}{{{\uplambda\:}}_{\mathrm{i}}}-{\mathrm{C}}_{3}{\upbeta\:}-{\mathrm{C}}_{4}){\mathrm{e}}^{-{\mathrm{C}}_{5}/{{\uplambda\:}}_{\mathrm{i}}}+{\mathrm{C}}_{6}{\uplambda\:}&\:&\:\end{array}$$

where $$\:{{\uplambda\:}}_{\mathrm{i}}={(\frac{1}{{\uplambda\:}+0.08{\upbeta\:}}-\frac{0.035}{{{\upbeta\:}}^{3}+1})}^{-1}$$,

And $C_{1} - C_{6}$are turbine-specific constants obtained experimentally.

The optimal tip-speed ratio at which maximum power occurs is given by:

3 $$\:\begin{array}{cccc}&\:{{\uplambda\:}}_{\mathrm{o}\mathrm{p}\mathrm{t}}=\frac{{{\upomega\:}}_{\mathrm{r}}\mathrm{R}}{{\mathrm{v}}_{\mathrm{w}}}&\:&\:\end{array}$$

where $\:{{\upomega\:}}_{\mathrm{r}}\:$is the rotor angular velocity (rad/s) and $\:\mathrm{R}$is the turbine blade radius (m).

#### PMSG modelling

The dynamic model of the PMSG in the synchronously rotating d_q_-frame is given by^42^:

4 $$\:\begin{array}{cccc}&\:{\mathrm{v}}_{\mathrm{d}}={\mathrm{R}}_{\mathrm{s}}{\mathrm{i}}_{\mathrm{d}}+{\mathrm{L}}_{\mathrm{d}}\frac{\mathrm{d}{\mathrm{i}}_{\mathrm{d}}}{\mathrm{d}\mathrm{t}}-{{\upomega\:}}_{\mathrm{e}}{\mathrm{L}}_{\mathrm{q}}{\mathrm{i}}_{\mathrm{q}}&\:&\:\end{array}$$

5 $$\:\begin{array}{cccc}&\:{\mathrm{v}}_{\mathrm{q}}={\mathrm{R}}_{\mathrm{s}}{\mathrm{i}}_{\mathrm{q}}+{\mathrm{L}}_{\mathrm{q}}\frac{\mathrm{d}{\mathrm{i}}_{\mathrm{q}}}{\mathrm{d}\mathrm{t}}+{{\upomega\:}}_{\mathrm{e}}({\mathrm{L}}_{\mathrm{d}}{\mathrm{i}}_{\mathrm{d}}+{{\uppsi\:}}_{\mathrm{f}})&\:&\:\end{array}$$

where

$$\:{\mathrm{v}}_{\mathrm{d}},{\mathrm{v}}_{\mathrm{q}}=$$ stator voltages (V),

$$\:{\mathrm{i}}_{\mathrm{d}},{\mathrm{i}}_{\mathrm{q}}$$= d_q_-axis currents (A),

$$\:{\mathrm{R}}_{\mathrm{s}}$$= stator resistance (Ω),

$$\:{\mathrm{L}}_{\mathrm{d}},{\mathrm{L}}_{\mathrm{q}}=$$ d_q_-axis inductances (H),

$\:{{\upomega\:}}_{\mathrm{e}}=\mathrm{p}{\hspace{0.17em}}{{\upomega\:}}_{\mathrm{r}}=$ electrical angular velocity (rad/s),

$\:{{\uppsi\:}}_{\mathrm{f}}=$ rotor permanent-magnet flux linkage (Wb).

The electromagnetic torque generated by the PMSG is expressed as:

6 $$\:\begin{array}{cccc}&\:{\mathrm{T}}_{\mathrm{e}}=\frac{3}{2}\mathrm{p}[{{\uppsi\:}}_{\mathrm{f}}{\mathrm{i}}_{\mathrm{q}}+({\mathrm{L}}_{\mathrm{d}}-{\mathrm{L}}_{\mathrm{q}}\left){\mathrm{i}}_{\mathrm{d}}{\mathrm{i}}_{\mathrm{q}}\right]&\:&\:\end{array}$$

where $\:\mathrm{p}\:$is the number of pole pairs.

### Boost converter modelling

To support controller design and accurate dynamic simulation, the Boost Converter is modeled using its switch-dependent state equations^43^.

Switch ON state ($\:\mathrm{S}=1$): When the switch is ON, the inductor is charged and the diode is reverse-biased.

7 $$\:\frac{\mathrm{d}{\mathrm{i}}_{\mathrm{L}}}{\mathrm{d}\mathrm{t}}=\frac{{\mathrm{V}}_{\mathrm{i}\mathrm{n}}}{\mathrm{L}}$$

8 $$\:\frac{\mathrm{d}{\mathrm{V}}_{\mathrm{d}\mathrm{c}}}{\mathrm{d}\mathrm{t}}=-\frac{{\mathrm{V}}_{\mathrm{d}\mathrm{c}}}{\mathrm{R}{\mathrm{C}}_{\mathrm{d}\mathrm{c}}}$$

Switch OFF state ($\:\mathrm{S}=0$): When the switch is OFF, the inductor releases energy to the load and the diode conducts.

9 $$\:\frac{\mathrm{d}{\mathrm{i}}_{\mathrm{L}}}{\mathrm{d}\mathrm{t}}=\frac{{\mathrm{V}}_{\mathrm{i}\mathrm{n}}-{\mathrm{V}}_{\mathrm{d}\mathrm{c}}}{\mathrm{L}}$$

10 $$\:\frac{\mathrm{d}{\mathrm{V}}_{\mathrm{d}\mathrm{c}}}{\mathrm{d}\mathrm{t}}=\frac{{\mathrm{i}}_{\mathrm{L}}-\frac{{\mathrm{V}}_{\mathrm{d}\mathrm{c}}}{\mathrm{R}}}{{\mathrm{C}}_{\mathrm{d}\mathrm{c}}}$$

Averaged State-Space Model Using duty cycle $\:\mathrm{D}$: Averaging (7)–(10) over one switching period gives:

11 $$\:\frac{\mathrm{d}{\mathrm{i}}_{\mathrm{L}}}{\mathrm{d}\mathrm{t}}=\mathrm{D}\left(\frac{{\mathrm{V}}_{\mathrm{i}\mathrm{n}}}{\mathrm{L}}\right)+(1-\mathrm{D})\left(\frac{{\mathrm{V}}_{\mathrm{i}\mathrm{n}}-{\mathrm{V}}_{\mathrm{d}\mathrm{c}}}{\mathrm{L}}\right)$$

12 $$\:\frac{\mathrm{d}{\mathrm{V}}_{\mathrm{d}\mathrm{c}}}{\mathrm{d}\mathrm{t}}=\mathrm{D}(-\frac{{\mathrm{V}}_{\mathrm{d}\mathrm{c}}}{\mathrm{R}{\mathrm{C}}_{\mathrm{d}\mathrm{c}}})+(1-\mathrm{D})\left(\frac{{\mathrm{i}}_{\mathrm{L}}-\frac{{\mathrm{V}}_{\mathrm{d}\mathrm{c}}}{\mathrm{R}}}{{\mathrm{C}}_{\mathrm{d}\mathrm{c}}}\right)$$

#### Compact state-space representation

Let,

13 $${\text{ }}x = \left[ {\begin{array}{*{20}c} {i_{L} } \\ {V_{{dc}} } \\ \end{array} } \right],u = V_{{in}}$$

Then,

14 $$\,\:\dot{x} = A\left( D \right)x + B\left( D \right)u$$

where,

15 $$\,\:A\left( D \right) = \left[ {\begin{array}{*{20}c} 0 & {\: - \frac{{1 - D}}{L}} \\ {\:\frac{{1 - D}}{{C_{{dc}} }}} & {\: - \frac{1}{{RC_{{dc}} }}} \\ \end{array} } \right]$$

and

16 $$\:\mathrm{B}\left(\mathrm{D}\right)=\left[\begin{array}{c}1/L\\\:0\end{array}\right]$$

These formulations ensure consistent variable notation and precise linkage between turbine dynamics, generator behavior, and converter control for accurate simulation of the PMSG–CHBI system.

### Modelling of five level cascaded H bridge inverter

**Fig. 2:**
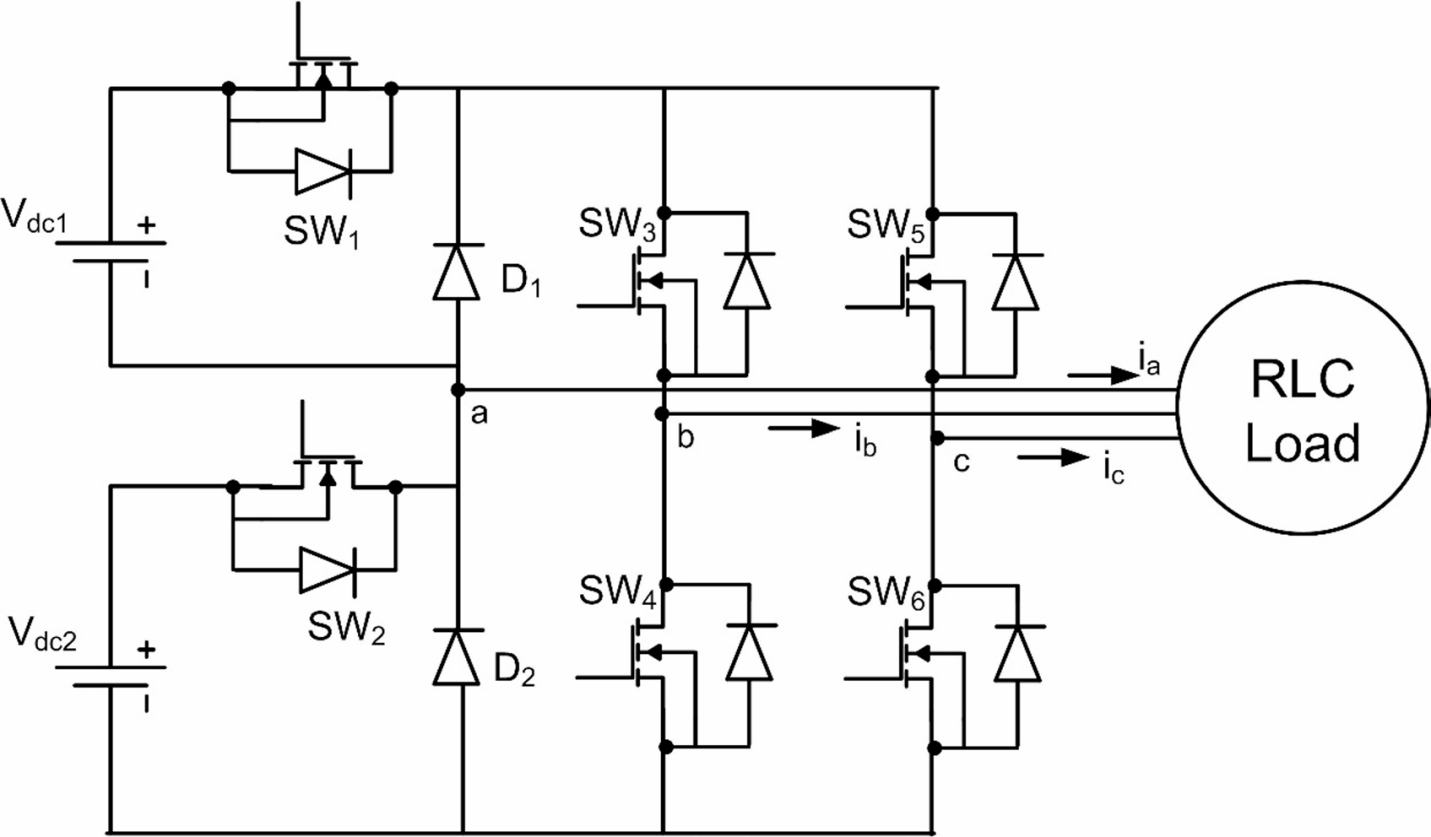
Structure of 5 level CHBI with 6 switches.

The proposed system employs two cascaded H-bridge (CHBI) cells powered by asymmetric DC sources to generate a five-level output waveform, as shown in Fig. 2^44^. This topology is selected for its modular structure and ability to achieve near-sinusoidal output with reduced harmonic distortion compared with lower-level inverter configurations. In PMSG-based wind systems, CHBIs are particularly suitable because isolated DC sources can be supplied directly from independent boost stages, ensuring straightforward voltage scaling and reduced device stress.

For modeling, each H-bridge cell produces three output states $\:(+{\mathrm{V}}_{\mathrm{d}\mathrm{c}},0,-{\mathrm{V}}_{\mathrm{d}\mathrm{c}})$, and the cascaded configuration generates five distinct voltage levels. The output phase voltage can be expressed as a summation of switching functions from the two cells. The switching angles corresponding to these voltage levels are later optimized using the GCRA algorithm, while the VRSTNN predictor compensates dynamic variations arising from unequal DC-link voltages. This modeling framework provides the foundation for harmonic analysis and controller integration presented in subsequent sections.

#### Switching States of the inverter

The five-level CHBI generates eight distinct switching modes corresponding to the voltage levels. 

**Fig. 3:**
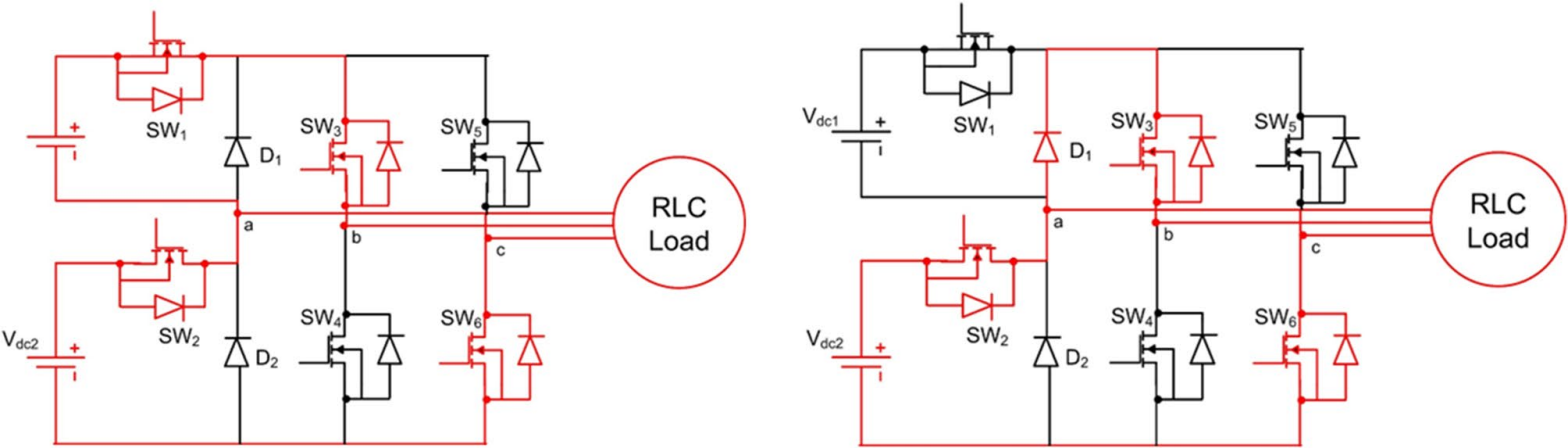
Analysis of (**a**) mode 1 (**b**) mode 2.

**Fig. 4:**
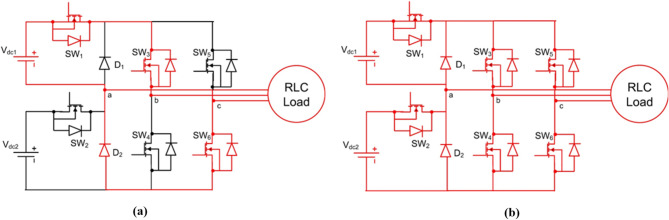
Analysis of (**a**) mode 3 (**b**) mode 4.

**Fig. 5:**
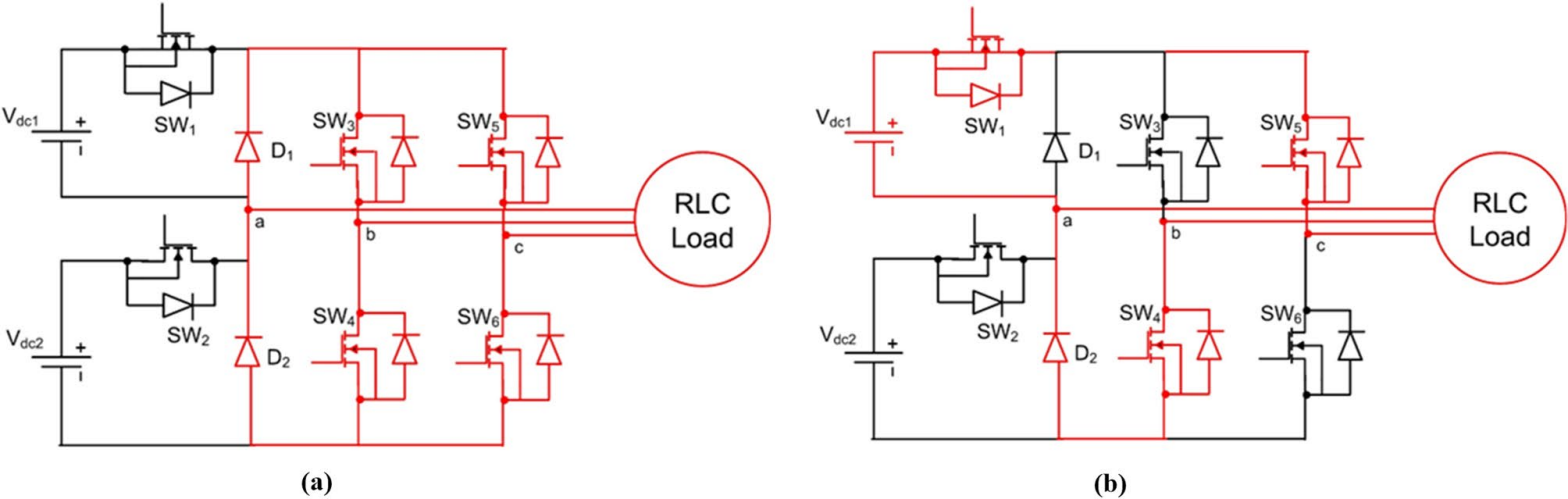
Analysis of (**a**) mode 5 (**b**) mode 6.

**Fig. 6:**
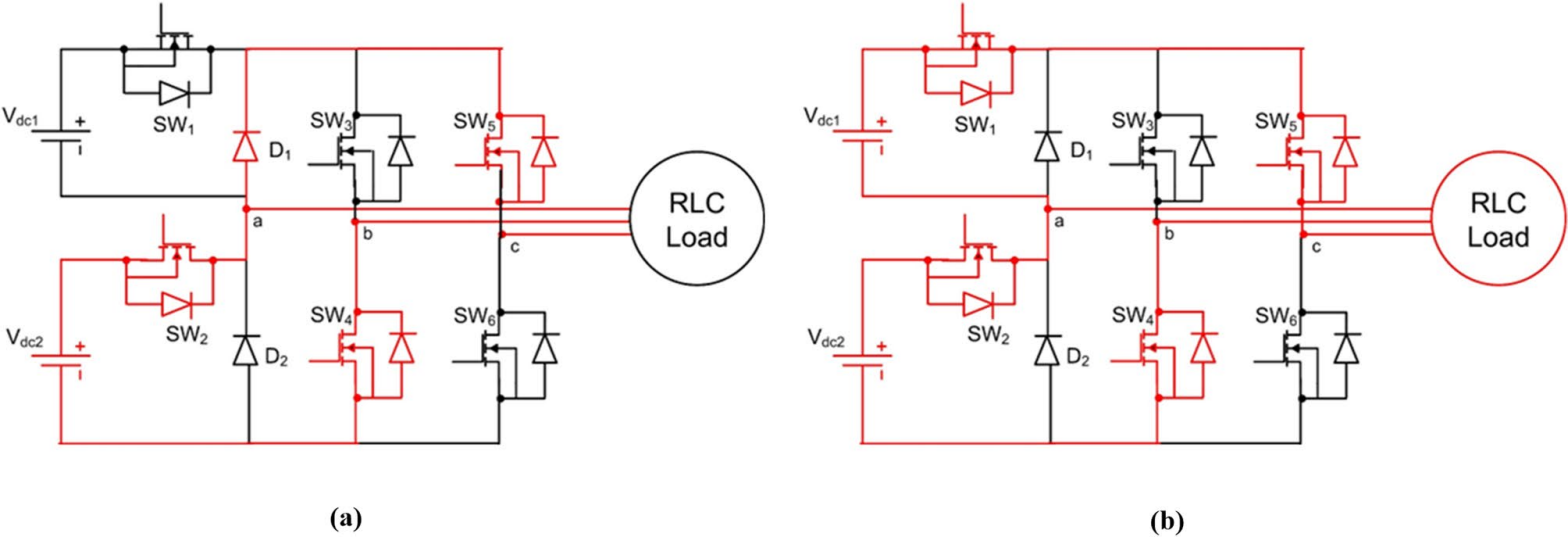
Analysis of mode 7 & mode 8.

**Fig. 7:**
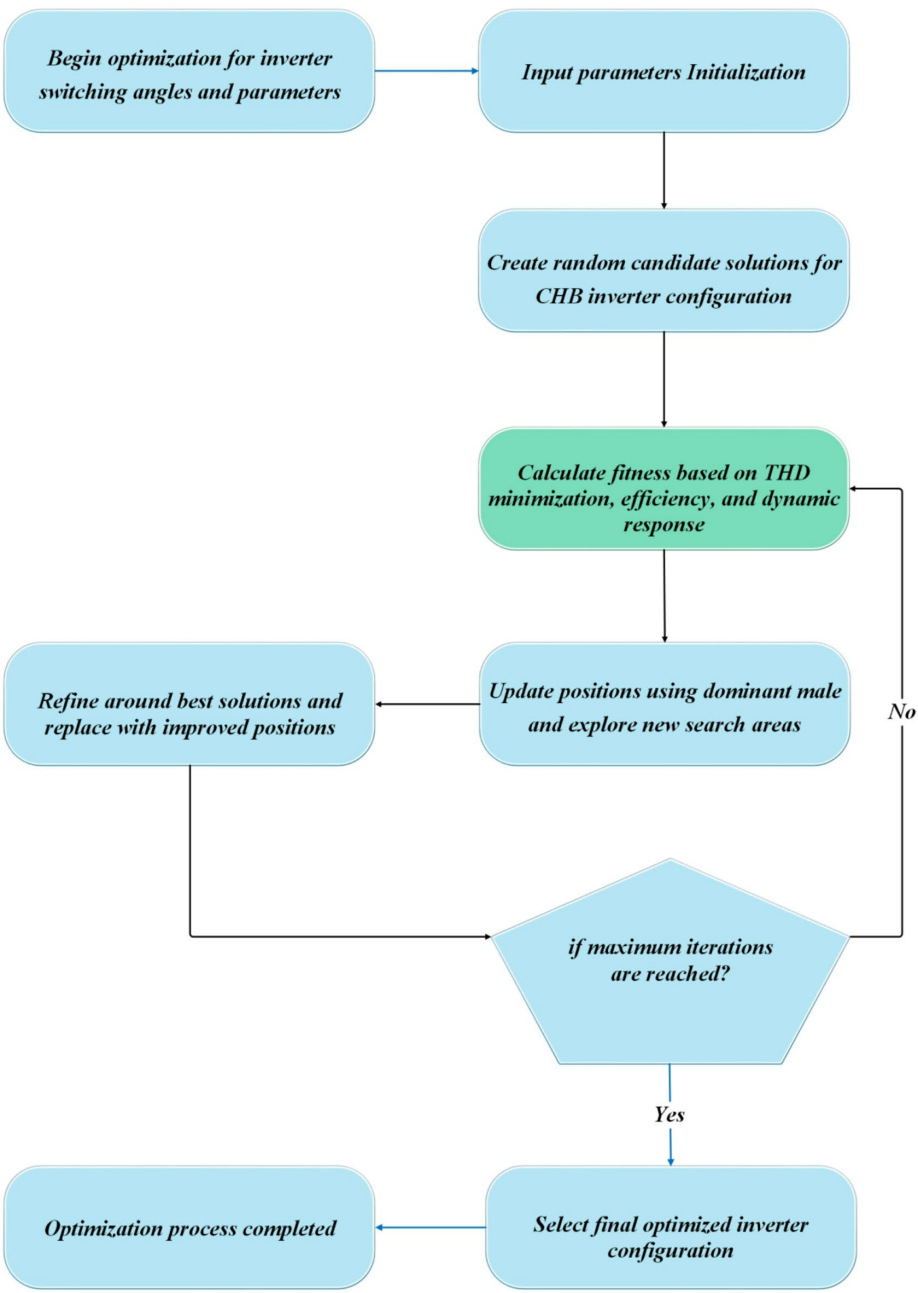
Flowchart of GCRA.

**Fig. 8:**
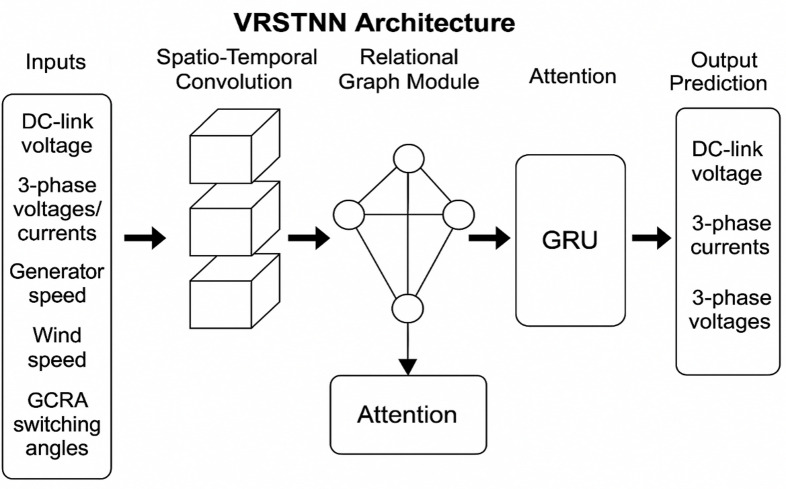
Architecture of VRSTNN for the proposed system.

**Fig. 9:**
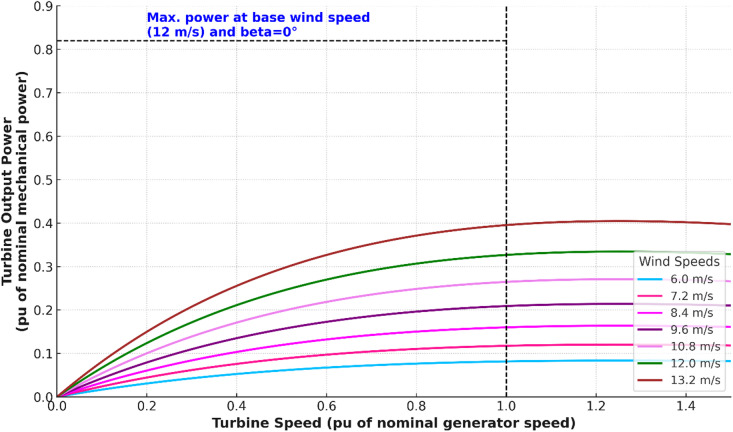
Analysis of Turbine Power. The Turbine power characteristics shows how output power increases with turbine speed across different wind velocities. The curves demonstrate that maximum power extraction occurs near the optimal tip-speed ratio, with higher wind speeds generating proportionally greater mechanical power while maintaining a consistent peak operating region. This confirms proper MPPT operation and accurate turbine modeling under varying wind conditions.

**Fig. 10:**
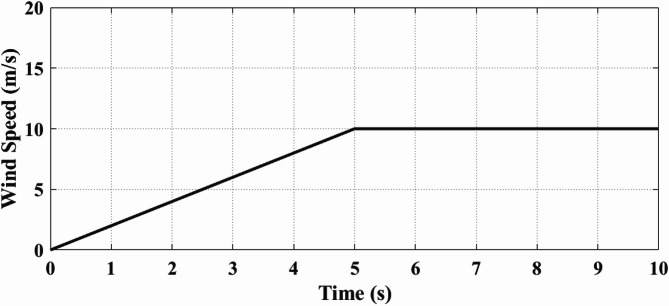
Analysis of Wind Speed which is used for dynamic testing, showing a gradual ramp from 0 to 10 m/s over the first 5 s followed by a steady region. This varying wind input enables assessment of the controller’s adaptability and the system’s transient response under realistic wind acceleration conditions.

$\:+{\mathrm{V}}_{\mathrm{d}\mathrm{c}1}+{\mathrm{V}}_{\mathrm{d}\mathrm{c}2}$, $\:+{\mathrm{V}}_{\mathrm{d}\mathrm{c}1}$, $\:+{\mathrm{V}}_{\mathrm{d}\mathrm{c}2}$, $\:0$, $\:-{\mathrm{V}}_{\mathrm{d}\mathrm{c}1}$, $\:-{\mathrm{V}}_{\mathrm{d}\mathrm{c}2}$, and $\:-({\mathrm{V}}_{\mathrm{d}\mathrm{c}1}+{\mathrm{V}}_{\mathrm{d}\mathrm{c}2})$. The switching states are summarized concisely in Table 1, showing the ON switches, diode conduction, and resulting output voltage. Modes 4 and 5 are both zero states, produced through different freewheeling or neutral conduction paths. Figures 3, 4, 5, 6 and 7 illustrate the corresponding modes of operation of the proposed inverter. The current flow in each operating mode is defined by the active conduction paths of the respective power switches, as illustrated in Figs. 3, 4, 5, 6, 7, 8, 9 and 10. The inverter produces V₀ = Vdc₁ + Vdc₂ in Mode 1, V₀ = Vdc₂ in Mode 2, V₀ = Vdc₁ in Mode 3, V₀ = 0 in Modes 4 and 5, V₀ = –Vdc₁ in Mode 6, V₀ = –Vdc₂ in Mode 7, and V₀ = –(Vdc₁ + Vdc₂) in Mode 8.

**Table 1:** Switching modes of the five-level CHBI inverter.

Mode	ON switches	Diode conduction	Output voltage	Description/function
1	SW1, SW2, SW3, SW6	None	+(V_dc1_+V_dc2_)	Full positive level (both cells active)
2	SW2, SW3, SW6	D1	+V_dc2_	Positive intermediate level (cell-2 active)
3	SW1, SW3, SW6	D2	+V_dc1_	Positive intermediate level (cell-1 active)
4	(All switches OFF)	Freewheeling diodes	0	Zero state A (neutral freewheeling)
5	SW4, SW5, SW6	Alternate freewheel	0	Zero state B (alternate zero path)
6	SW1, SW4, SW5	D2	-V_dc1_	Negative intermediate level (cell-1 negative)
7	SW2, SW4, SW5	D1	-V_dc2_	Negative intermediate level (cell-2 negative)
8	SW1, SW2, SW4, SW5	None	-(V_dc1+_V_dc2_)	Full negative level (both cells active)

##### Mode 1 & 2

See Fig. 3.

##### Mode 3 & 4

See Fig. 4.

##### Mode 5 & 6

See Fig. 5.

##### Mode 7 & 8

See Fig. 6.

### Objective function

The objective function of this study is to minimize the THD of the output voltage waveform produced by the multilevel inverter. THD is mathematically defined as the ratio of the root mean square value of all harmonic components to the root mean square value of the fundamental component. The objective function can therefore be expressed in equations:

17 $$\:\mathrm{T}\mathrm{H}\mathrm{D}=100\times\:\frac{\sqrt{{\sum\:}_{\mathrm{n}=\mathrm{3,5},...,49}{\mathrm{V}}_{\mathrm{n}}^{2}}}{{\mathrm{V}}_{1}}$$

18 $$\:{\mathrm{F}}_{\mathrm{o}\mathrm{b}\mathrm{j}}=\underset{\left\{{{\upalpha\:}}_{\mathrm{k}}\right\}}{\mathrm{m}\mathrm{i}\mathrm{n}}\left(\mathrm{T}\mathrm{H}\mathrm{D}\right)$$

Where $\:{\mathrm{F}}_{\mathrm{o}\mathrm{b}\mathrm{j}}$represents the optimization objective, which is to minimize this distortion. The parameter $\:{{\upalpha\:}}_{\mathrm{k}}$implies the switching angle of the $\:{\mathrm{k}}^{\mathrm{t}\mathrm{h}}$ inverter cell. The $\:\mathrm{n}$ represents the harmonic order, which takes odd values such as$\:\mathrm{1,3},5,...,49$. The term $\:{\mathrm{V}}_{\mathrm{n}}$indicates the amplitude of the $\:{\mathrm{n}}^{\mathrm{t}\mathrm{h}}$harmonic component of the output voltage, and $\:{\mathrm{V}}_{1}$​ represents the amplitude of the fundamental (first harmonic) component of the output voltage. The harmonic components of the inverter output voltage are obtained from the following relation:

19 $$\:{\mathrm{V}}_{\mathrm{n}}=\frac{4}{\mathrm{n}{\uppi\:}}{\sum\:}_{\mathrm{k}=1}^{\mathrm{s}}{\mathrm{V}}_{\mathrm{d}\mathrm{c},\mathrm{k}}\mathrm{cos}\left(\mathrm{n}{{\upalpha\:}}_{\mathrm{k}}\right)$$

where $\:\mathrm{s}$denotes the number of inverter cells per phase, $\:{\mathrm{V}}_{\mathrm{d}\mathrm{c},\mathrm{k}}$ stands for the DC source voltage of $\:{\mathrm{k}}^{\mathrm{t}\mathrm{h}}$ inverter cell, $\:{\uppi\:}$ is a mathematical constant, and $\:\mathrm{cos}\left(\mathrm{n}{{\upalpha\:}}_{\mathrm{k}}\right)$ denotes the cosine function relating the harmonic order to the switching angle.

Equations (10)–(12) clearly define THD and establish the optimization objective to determine the optimal switching angles $\:{{\upalpha\:}}_{\mathrm{k}}$such that the THD of the inverter output voltage is minimized.

### THD optimization problem constraints

The switching-angle optimization aims to minimize THD while satisfying the inverter’s operational limits. The constraints are formulated as follows.

#### Switching-angle ordering constraint

For a five-level CHBI, the switching angles must satisfy:

20 $$0 < \alpha _{1} < \alpha _{2} < \alpha _{3} < \frac{\pi }{2}$$

This enforces proper quarter-wave symmetry and ensures that higher-level switching occurs later in the waveform.

#### Fundamental component constraint (modulation index requirement)

To generate the required fundamental output voltage $\:{\mathrm{V}}_{1}^{\mathrm{*}}$, the switching angles must satisfy:

21 $$V_{1}^{*} = \frac{4}{{\pi \:}}[V_{{dc1}} \cos (\alpha \:_{1} ) + V_{{dc2}} \cos (\alpha \:_{2} ) + V_{{dc1}} \cos (\alpha \:_{3} )]$$

Or equivalently:

22 $$M = \frac{{V_{1}^{*} }}{{V_{{1,\max }} }}$$

where

- $\:\mathrm{M}$is the modulation index,
- $\:{\mathrm{V}}_{1,\mathrm{max}}$is the maximum achievable fundamental component for the given DC sources.

This ensures that THD minimization does not compromise the required output voltage.

#### Harmonic constraints

Unwanted low-order harmonics must be minimized or eliminated:

23 $$\:H_{k} \left( {\alpha \:_{i} } \right) \approx \:0k = 5,7,11,13, \ldots$$

where $\:{\mathrm{H}}_{\mathrm{k}}$ is the k-th harmonic component computed from the Fourier series.

#### Device and operating limits

The optimization must keep switching angles within safe device-operating boundaries:

24 $$\:\alpha \:_{i} \in \:[\alpha \:_{{\min }} ,\alpha \:_{{\max }} ]$$

And maintain DC-link constraints:

$$\:{\mathrm{V}}_{\mathrm{d}\mathrm{c}1},{\hspace{0.17em}}{\mathrm{V}}_{\mathrm{d}\mathrm{c}2}>0$$

$$\:{\mathrm{V}}_{\mathrm{d}\mathrm{c}1}\ne\:{\mathrm{V}}_{\mathrm{d}\mathrm{c}2}\left(\mathrm{asymmetric\:configuration}\right)$$

These constraints ensure physical realizability in a CHBI topology.

## Proposed GCRA-VRSTNN method for THD minimization and performance optimization

The planned GCRA-VRSTNN process for minimizing THD hybridizes GCRA and VRSTNN in order to improve the performance of the CHBI-assisted PMSG wind system. The GCRA is used to optimize the switching angles and operation parameters for harmonic reduction, while the VRSTNN is used to model the dynamic nonlinear operation of the system and provide adaptive control for different wind conditions. The hybridized process achieves a significant reduction in THD and also improves performance and system stability.

### Optimization using greater cane rat algorithm

GCRA is a metaheuristic optimization technique inspired by the foraging and social behavior of greater cane rats in their natural habitat^45^. Within the context of energy systems, GCRA imitates how individual agents explore and exploit their environment to locate optimal resources by mapping this behavior to the search for optimal inverter switching angles and operational parameters. In this study, each cane rat agent represents a candidate configuration of the CHBI, including switching patterns, modulation indices, and relevant performance parameters. GCRA achieves adaptive coordination between global exploration and local exploitation by regulating agent movement, social interactions, and fitness-based selection in each iteration. This balance allows the algorithm to efficiently navigate complex optimization landscapes, targeting objectives such as THD minimization, efficiency enhancement, and dynamic response improvement. A notable advantage of GCRA is its population diversity maintenance, which prevents premature convergence and ensures robust search capabilities across nonlinear and multi-dimensional solution spaces. Unlike conventional optimization techniques that may get trapped in local minima, GCRA adapts its search strategy dynamically across iterations, refining inverter operational settings to achieve optimal performance. The Greater Cane Rat Algorithm (GCRA) models the exploratory and exploitative behavior of agents (rats) using dynamically adjusted control coefficients. Each rat represents a candidate solution vector of inverter switching parameters $\:{\mathrm{X}}_{\mathrm{i}}=[{{\upalpha\:}}_{1},{{\upalpha\:}}_{2},...,{{\upalpha\:}}_{\mathrm{m}}]$. The detailed procedure of GCRA implementation is described in the following steps:

#### Step 1: initialization

Each agent is initialized randomly within the parameter search space:

25 $$\:\begin{array}{cccc}&\:{\mathrm{X}}_{\mathrm{i}}^{\left(0\right)}={\mathrm{X}}_{\mathrm{min}}+\mathrm{r}\times\:({\mathrm{X}}_{\mathrm{max}}-{\mathrm{X}}_{\mathrm{min}}),&\:&\:\mathrm{(25)}\end{array}$$

where$\:{\mathrm{X}}_{\mathrm{i}}^{\left(0\right)}$= initial position of the $\:\mathrm{i}$-th agent,

$$\:\mathrm{r}\in\:\left[\mathrm{0,1}\right]=$$ random vector,

$\:{\mathrm{X}}_{\mathrm{min}},{\mathrm{X}}_{\mathrm{max}}$= lower and upper parameter bounds.

The population size is $\:\mathrm{N}$, and each vector dimension $\:\mathrm{m}$corresponds to one control variable (e.g., switching angle, modulation index).

#### Step 2: fitness evaluation

The fitness of each agent is computed using the objective function — minimization of Total Harmonic Distortion (THD):

26 $$\:\begin{array}{cccc}&\:\mathrm{f}\left({\mathrm{X}}_{\mathrm{i}}\right)=\mathrm{THD}\left({\mathrm{X}}_{\mathrm{i}}\right)=\sqrt{\frac{\sum\:_{\mathrm{n}=2}^{{\mathrm{N}}_{\mathrm{h}}}{\mathrm{V}}_{\mathrm{n}}^{2}}{{\mathrm{V}}_{1}^{2}}}&\:&\:\end{array}$$

where $\:{\mathrm{V}}_{\mathrm{n}}\:$denotes the $\:\mathrm{n}$-th harmonic component and $\:{\mathrm{V}}_{1}$the fundamental voltage amplitude.

The agent with the lowest fitness $\:{\mathrm{f}}_{\mathrm{best}}$is designated as the dominant male (leader).

#### Step 3: exploration phase (diversification)

The exploration stage models random foraging and shelter-searching behavior:

27 $$\:\begin{array}{cccc}&\:{\mathrm{X}}_{\mathrm{i}}^{(\mathrm{t}+1)}={\mathrm{X}}_{\mathrm{i}}^{\left(\mathrm{t}\right)}+{\upgamma\:}{\hspace{0.17em}}{\mathrm{r}}_{1}{\hspace{0.17em}}({\mathrm{X}}_{\mathrm{best}}^{\left(\mathrm{t}\right)}-{\mathrm{X}}_{\mathrm{i}}^{\left(\mathrm{t}\right)})+\mathrm{S}\left(\mathrm{t}\right){\hspace{0.17em}}{\mathrm{r}}_{2}{\hspace{0.17em}}({\mathrm{X}}_{\mathrm{j}}^{\left(\mathrm{t}\right)}-{\mathrm{X}}_{\mathrm{k}}^{\left(\mathrm{t}\right)})&\:&\:\end{array}$$

where

$$\:{\upgamma\:}\in\:\left(\mathrm{0,1}\right)=$$ diversification factor,

$$\:{\mathrm{r}}_{1},{\mathrm{r}}_{2}\in\:\left[\mathrm{0,1}\right]=$$ random coefficients,

$\:{\mathrm{X}}_{\mathrm{j}},{\mathrm{X}}_{\mathrm{k}}=$ random distinct agents,

$\:\mathrm{S}\left(\mathrm{t}\right)={\mathrm{S}}_{0}{\hspace{0.17em}}{\mathrm{e}}^{-\mathrm{t}/{\mathrm{T}}_{\mathrm{m}\mathrm{a}\mathrm{x}}}$— seasonality adaptation function controlling exploration intensity.

If the new position yields a better fitness:

28 $$\:\begin{array}{cccc}&\:\mathrm{f}\left({\mathrm{X}}_{\mathrm{i}}^{(\mathrm{t}+1)}\right)<\mathrm{f}\left({\mathrm{X}}_{\mathrm{i}}^{\left(\mathrm{t}\right)}\right)\Rightarrow\:{\mathrm{X}}_{\mathrm{i}}^{\left(\mathrm{t}\right)}={\mathrm{X}}_{\mathrm{i}}^{(\mathrm{t}+1)}&\:&\:\end{array}$$

This mechanism prevents premature convergence and maintains population diversity.

#### Step 4: intensification phase (exploitation)

In the intensification phase, agents concentrate around the best-known position to refine the search:

29 $$X_{i}^{{(t + 1)}} = X_{{best}}^{{\left( t \right)}} + \delta \:r_{3} (X_{f}^{{\left( t \right)}} - X_{i}^{{\left( t \right)}} ),$$

where$\:{\updelta\:}\in\:\left(\mathrm{0,1}\right)$= breeding (intensification) factor,

$$\:{\mathrm{r}}_{3}\in\:\left[\mathrm{0,1}\right]$$= random coefficient,

$\:{\mathrm{X}}_{\mathrm{f}}^{\left(\mathrm{t}\right)}$= randomly selected female agent representing a local optimum.

The exploitation strength is further controlled by:

30 $$\:\begin{array}{cccc}&\:{\upeta\:}\left(\mathrm{t}\right)={{\upeta\:}}_{0}(1-\frac{\mathrm{t}}{{\mathrm{T}}_{\mathrm{m}\mathrm{a}\mathrm{x}}}),&\:&\:\end{array}$$

where $\:{{\upeta\:}}_{0}$ is the initial exploitation weight, gradually reduced with iteration count $\:\mathrm{t}$.

The updated position is accepted when:

31 $$\:\begin{array}{cccc}&\:\mathrm{f}\left({\mathrm{X}}_{\mathrm{i}}^{(\mathrm{t}+1)}\right)<\mathrm{f}\left({\mathrm{X}}_{\mathrm{i}}^{\left(\mathrm{t}\right)}\right)\Rightarrow\:{\mathrm{X}}_{\mathrm{i}}^{\left(\mathrm{t}\right)}={\mathrm{X}}_{\mathrm{i}}^{(\mathrm{t}+1)}&\:&\:\end{array}$$

#### Step 5: seasonal adaptation and dynamic balancing

The GCRA transitions smoothly between exploration and exploitation using the adaptive balancing coefficient:

32 $$\:\begin{array}{cccc}&\:{\upvarphi\:}\left(\mathrm{t}\right)=\frac{1}{1+{\mathrm{e}}^{-\mathrm{a}(\mathrm{t}-{\mathrm{T}}_{\mathrm{m}\mathrm{a}\mathrm{x}}/2)}}&\:&\:\end{array}$$

where $\:\mathrm{a}$is a scaling constant controlling transition sharpness.

The overall update rule that integrates both phases is:

33 $$\:\begin{array}{cccc}&\:{\mathrm{X}}_{\mathrm{i}}^{(\mathrm{t}+1)}=(1-{\upvarphi\:}(\mathrm{t}\left)\right){\hspace{0.17em}}{\mathrm{X}}_{\mathrm{explore}}^{\left(\mathrm{t}\right)}+{\upvarphi\:}\left(\mathrm{t}\right){\hspace{0.17em}}{\mathrm{X}}_{\mathrm{exploit}}^{\left(\mathrm{t}\right)}&\:&\:\end{array}$$

#### Step 6: termination criterion

The algorithm terminates when the stopping condition is met:

34 $$\:\begin{array}{cccc}&\:\mathrm{m}\mathrm{i}\mathrm{n}\left(\mathrm{f}\right({\mathrm{X}}_{\mathrm{i}}\left)\right)<{\upepsilon\:}\mathrm{or}\mathrm{t}\ge\:{\mathrm{T}}_{\mathrm{m}\mathrm{a}\mathrm{x}}&\:&\:\end{array}$$

where $\:{\upepsilon\:}$is the desired convergence threshold and $\:{\mathrm{T}}_{\mathrm{m}\mathrm{a}\mathrm{x}}$is the maximum number of iterations.

The best solution $\:{\mathrm{X}}_{\mathrm{best}}$gives the optimal inverter switching angles and operational parameters:

35 $$\:\begin{array}{cccc}&\:{\mathrm{X}}_{\mathrm{opt}}=\mathrm{a}\mathrm{r}\mathrm{g}\underset{{\mathrm{X}}_{\mathrm{i}}\in\:{\Omega\:}}{\mathrm{m}\mathrm{i}\mathrm{n}}\mathrm{f}\left({\mathrm{X}}_{\mathrm{i}}\right)&\:&\:\end{array}$$

#### Advantages of GCRA over existing optimizers

Compared to established metaheuristics such as GA, PSO, and RPOA, GCRA offers several benefits:

1. Exploration–Exploitation Balance: Seasonal adaptation enables smooth transitions between global exploration and local exploitation, thereby reducing the risk of premature convergence that is common in GA and PSO.
2. Population Diversity: By simulating multiple sheltering and foraging patterns, GCRA maintains diversity in the solution pool, unlike GA, which may converge prematurely.
3. Low Parameter Dependence: GCRA requires fewer control parameters, making it easier to tune compared to GA (crossover/mutation) or RPOA (transformer hyperparameters).
4. Robustness in Nonlinear Search Spaces: The hybridized update strategy (peer influence + leader attraction) allows GCRA to effectively handle the nonlinear optimization of inverter switching angles.

These features make GCRA particularly well-suited for optimizing CHBI operation in PMSG-based wind systems, where the objective function involves minimizing THD under dynamic and nonlinear conditions.

###  Prediction using Visual-Relational Spatio-Temporal Neural Network

The Visual-Relational Spatio-Temporal Neural Network (VRSTNN) is a deep learning model specifically designed to capture both spatial and temporal dependencies in complex energy systems^46^. In this study, VRSTNN is employed as shown in Fig. 8 to accurately model and predict the dynamic behavior of PMSG-based wind turbine (WT) systems interfaced with a three-phase five-level CHBI. The network generates time-variant spatio-temporal feature maps that represent interactions among generator dynamics, inverter switching states, and voltage outputs over time. By leveraging variational inference and spatio-temporal convolutions, VRSTNN learns both localized variations (e.g., voltage ripple at individual phases) and system-wide patterns (e.g., harmonic propagation across the inverter). An attention-based mechanism further enhances accuracy by dynamically weighting the significance of different temporal events and spatial nodes, thereby improving the fidelity of forecasts.

Compared to conventional Artificial Neural Networks (ANNs), Convolutional Neural Networks (CNNs), or recurrent models such as RNNs and LSTMs, VRSTNN introduces several critical improvements that make it particularly suited for wind energy applications:

- Spatio-temporal feature learning: While ANNs and RNNs treat inputs primarily as sequences, VRSTNN integrates convolutional operations with relational graph-based updates, enabling it to capture both local switching variations and global interdependencies within the wind energy system.
- Relation-aware modeling: Unlike CNNs that focus on localized spatial patterns, VRSTNN encodes explicit relationships between system elements (generator, rectifier, inverter, and load) using relational graph aggregation, ensuring that dynamic interdependencies are preserved.
- Attention-driven prediction: The network incorporates attention mechanisms to prioritize critical features such as sudden wind gusts, torque changes, or harmonic distortions, leading to more robust control decisions.
- Robustness to nonlinear dynamics: In highly nonlinear environments, such as PMSG–CHBI systems, VRSTNN demonstrates better adaptability and generalization compared to ANNs, which may overfit, or CNNs, which require very large datasets.

These properties make VRSTNN particularly well-suited for spatio-temporal modeling in PMSG-based CHBI wind energy systems, where accurate prediction of system behavior under fluctuating wind and load conditions is essential for minimizing THD, reducing voltage ripples, and ensuring dynamic stability.

The Visual-Relational Spatio-Temporal Neural Network (VRSTNN) is adapted here for multisignal feature learning from the inverter–generator system rather than for image analysis. The term visual denotes the two-dimensional tensor representation of electrical quantities over time (analogous to a dynamic image), enabling convolutional learning on structured temporal data.

The VRSTNN receives the following input features at each sampling instant $\:\mathrm{t}$: $\:\mathrm{X}\left(\mathrm{t}\right)=\left[{\mathrm{V}}_{\mathrm{d}\mathrm{c}}\right(\mathrm{t}),{\hspace{0.17em}}{\mathrm{I}}_{\mathrm{a}}(\mathrm{t}),{\hspace{0.17em}}{\mathrm{I}}_{\mathrm{b}}(\mathrm{t}),{\hspace{0.17em}}{\mathrm{I}}_{\mathrm{c}}(\mathrm{t}),{\hspace{0.17em}}{\mathrm{V}}_{\mathrm{a}}(\mathrm{t}),{\hspace{0.17em}}{\mathrm{V}}_{\mathrm{b}}(\mathrm{t}),{\hspace{0.17em}}{\mathrm{V}}_{\mathrm{c}}(\mathrm{t}),{\hspace{0.17em}}{{\upomega\:}}_{\mathrm{r}}(\mathrm{t}),{\hspace{0.17em}}{\mathrm{v}}_{\mathrm{w}}(\mathrm{t}),{\hspace{0.17em}}{\upalpha\:}(\mathrm{t}\left)\right]$. 

where

$\:{\mathrm{V}}_{\mathrm{d}\mathrm{c}}=$ DC-link voltage,

$\:{\mathrm{I}}_{\mathrm{a},\mathrm{b},\mathrm{c}}=$ three-phase currents,

$\:{\mathrm{V}}_{\mathrm{a},\mathrm{b},\mathrm{c}}=$ three-phase voltages,

$$\:{{\upomega\:}}_{\mathrm{r}}$$= generator speed,

$$\:{\mathrm{v}}_{\mathrm{w}}=$$ wind velocity,

$\:{\upalpha\:}$= inverter switching angles from GCRA.

The target output is the predicted inverter state vector:

36 $$\:\mathbf{Y}(\mathrm{t}+{\uptau\:})=\left[{\widehat{\mathrm{V}}}_{\mathrm{o}}\right(\mathrm{t}+{\uptau\:}),{\hspace{0.17em}}{\widehat{\mathrm{I}}}_{\mathrm{o}}(\mathrm{t}+{\uptau\:}),{\hspace{0.17em}}\widehat{\mathrm{THD}}(\mathrm{t}+{\uptau\:}\left)\right]$$

where $\:{\uptau\:}$is the prediction horizon.

#### Mathematical model

Each VRSTNN layer captures spatial and temporal dependencies between these coupled parameters.

#### Spatio-temporal convolution layer

37 $$\:\begin{array}{cccc}&\:{\mathrm{H}}_{\mathrm{s}}^{\left(\mathrm{l}\right)}={\upsigma\:}{\hspace{0.17em}}({\mathrm{W}}_{\mathrm{s}}^{\left(\mathrm{l}\right)}\mathrm{*}{\mathrm{X}}^{(\mathrm{l}-1)}+{\mathrm{b}}_{\mathrm{s}}^{\left(\mathrm{l}\right)})&\:&\:\mathrm{(37)}\end{array}$$

where

$\:{\mathrm{W}}_{\mathrm{s}}^{\left(\mathrm{l}\right)}$= learnable 2D convolutional kernel,

$\:\mathrm{*}$ = convolution operator across both feature and temporal axes,

$$\:{\upsigma\:}\left(\cdot\:\right)=$$ ReLU activation.

This operation captures intra-phase correlations and temporal voltage-current coupling.

#### Relational graph update

The network then models the dynamic relationships among CHBI subcomponents (generator → rectifier → DC link → inverter → load) as a graph $\:\mathcal{G}=(\mathcal{V},\mathcal{E})$, where each node represents a subsystem.

38 $$\:\begin{array}{cccc}&\:{\mathrm{H}}_{\mathrm{r}}^{\left(\mathrm{l}\right)}\left({\mathrm{v}}_{\mathrm{i}}\right)={\upvarphi\:}(\sum\:_{{\mathrm{v}}_{\mathrm{j}}\in\:\mathcal{N}\left({\mathrm{v}}_{\mathrm{i}}\right)}{\mathrm{W}}_{\mathrm{r}}^{\left(\mathrm{l}\right)}{\hspace{0.17em}}{\mathrm{H}}_{\mathrm{s}}^{\left(\mathrm{l}\right)}\left({\mathrm{v}}_{\mathrm{j}}\right)+{\mathrm{b}}_{\mathrm{r}}^{\left(\mathrm{l}\right)})&\:&\:\mathrm{(38)}\end{array}$$

where

$\:{\mathrm{W}}_{\mathrm{r}}^{\left(\mathrm{l}\right)}=$relational weight matrix,

$N(v_i)$ = neighboring nodes of v_i_

$\:{\upvarphi\:}=$ non-linear activation (LeakyReLU).

This step captures inter-component coupling, such as how variations in wind torque affect inverter voltage ripple.

#### Temporal recurrence

To track system evolution, hidden states are updated using gated recurrent units (GRUs).

39 $$\:\begin{array}{cccc}&\:{\mathrm{h}}_{\mathrm{t}}=(1-{\mathrm{z}}_{\mathrm{t}}){\mathrm{h}}_{\mathrm{t}-1}+{\mathrm{z}}_{\mathrm{t}}{\hspace{0.17em}}\mathrm{t}\mathrm{a}\mathrm{n}\mathrm{h}{\hspace{0.17em}}({\mathrm{W}}_{\mathrm{h}}{\mathrm{H}}_{\mathrm{r}}^{\left(\mathrm{l}\right)}+{\mathrm{U}}_{\mathrm{h}}({\mathrm{r}}_{\mathrm{t}}\odot\:{\mathrm{h}}_{\mathrm{t}-1}\left)\right)&\:&\:\mathrm{(39)}\end{array}$$

where $\:{\mathrm{z}}_{\mathrm{t}}$and $\:{\mathrm{r}}_{\mathrm{t}}$are update and reset gates defined by:

$$\:\begin{array}{cccc}&\:{\mathrm{z}}_{\mathrm{t}}={\upsigma\:}({\mathrm{W}}_{\mathrm{z}}{\mathrm{H}}_{\mathrm{r}}^{\left(\mathrm{l}\right)}+{\mathrm{U}}_{\mathrm{z}}{\mathrm{h}}_{\mathrm{t}-1}),{\mathrm{r}}_{\mathrm{t}}={\upsigma\:}({\mathrm{W}}_{\mathrm{r}}{\mathrm{H}}_{\mathrm{r}}^{\left(\mathrm{l}\right)}+{\mathrm{U}}_{\mathrm{r}}{\mathrm{h}}_{\mathrm{t}-1})&\:&\:\mathrm{(40)}\end{array}$$

#### Prediction layer

The final output layer maps the hidden state to predicted operating variables.

$$\:\begin{array}{cccc}&\:\widehat{\mathrm{Y}}(\mathrm{t}+{\uptau\:})={\mathrm{W}}_{\mathrm{o}}{\mathrm{h}}_{\mathrm{t}}+{\mathrm{b}}_{\mathrm{o}}&\:&\:\mathrm{(41)}\end{array}$$

where $\:{\mathrm{W}}_{\mathrm{o}}$and $\:{\mathrm{b}}_{\mathrm{o}}$are output weights and biases.

#### Interaction between GCRA and VRSTNN

The proposed framework operates on two distinct timescales. First, GCRA performs an offline (or slow supervisory) optimization to compute the optimal switching-angle set that minimizes low-order harmonics and satisfies modulation constraints. These optimized angles remain fixed during the real-time operation of the inverter. In contrast, VRSTNN functions entirely online, predicting short-term dynamic variations such as DC-link fluctuations, transient voltage deviations, and ripple formation. The VRSTNN output provides dynamic correction signals (e.g., duty-cycle or pulse-width adjustments) but does not modify or rerun the GCRA optimization loop. This two-layer structure ensures that the inverter benefits from globally optimal switching angles while still adapting rapidly to nonlinear and time-varying operating conditions.

### Parameter tuning for GCRA

The performance of GCRA depends on a small set of parameters, including population size (N), diversification factor (γ), breeding factor (δ), and the seasonality adaptation coefficient (S(t)). Initial ranges for these parameters were taken from the literature, and then refined through a parametric sweep experiment on the PMSG–CHBI test system. The best convergence and lowest THD were achieved with *N* = 40, γ = 0.8, δ = 0.6, and S(t) = 0.5. Sensitivity analysis was performed by perturbing each parameter ± 20% around its tuned value. Results showed that THD variation remained within ± 0.3%, confirming that the GCRA configuration achieved the objective defined.

### Parameter tuning for VRSTNN

For VRSTNN, hyperparameters such as learning rate, number of convolutional layers, hidden state dimension, and attention window size were tuned using grid search and validation on simulated wind datasets. The final configuration used a learning rate of 0.001, three spatio-temporal convolutional layers, a hidden state size of 128, and an attention window of 10 time steps. Sensitivity analysis was performed by varying each hyperparameter individually while keeping others fixed. The results indicated that VRSTNN performance (measured in prediction accuracy and THD reduction) remained stable for ± 15% changes in hidden state size and attention window, while the learning rate required stricter tuning. This confirms the resilience of VRSTNN to moderate hyperparameter variations under fluctuating wind conditions.

## Result and discussion

This section uses simulation results to demonstrate effectiveness of suggested method. In WT system with a 3 phase 5 level CHBI, the VRSTNN–GCRA framework is introduced. The suggested VRSTNN–GCRA approach is evaluated against conventional methods such as ANN, RERNN-LSE, RPOA-DTRN, GA-PSO, and CNNs, and it is implemented on the MATLAB/Simulink platform. The simulations were performed using MATLAB/Simulink with the parameters summarized here to ensure reproducibility. The PMSG is rated at 2.5 kW, 400 V, with $\:{\mathrm{L}}_{\mathrm{d}}=5.2\mathrm{\:mH}$, $\:{\mathrm{L}}_{\mathrm{q}}=4.8\mathrm{\:mH}$, Rs=0.78 Ohms and $\:{{\uplambda\:}}_{\mathrm{f}}=0.175\mathrm{\:Wb}$. The five-level CHBI operates with two isolated DC sources $\:{\mathrm{V}}_{\mathrm{d}\mathrm{c}1}={\mathrm{V}}_{\mathrm{d}\mathrm{c}2}=110\mathrm{\:V}$, producing a combined 220 V DC-link after the boost converter. The switching frequency is set to 10 kHz and the controller sampling time to 50 µs. The wind-speed profile varies from 6 to 12 m/s to test dynamic adaptability. For optimization, GCRA uses a population size of 30, maximum iterations of 50, and dynamic search coefficients, while VRSTNN employs three spatio-temporal layers with a learning rate of 0.001 and a training window of 200 samples. In this work, turbine power results are expressed in per unit (pu) with respect to the rated turbine power, while torque, voltage, and current are shown in absolute units (N_m_, V, A). To ensure statistical robustness, all simulations were repeated for 10 independent runs with randomized initial conditions. Reported performance metrics including THD, voltage ripple, power loss, and dynamic response time—represent the mean value across these runs, while the corresponding standard deviations quantify run-to-run variability. The proposed GCRA–VRSTNN approach demonstrated low variance, with THD fluctuating within ± 0.04%, voltage ripple within ± 0.12%, and dynamic response variations less than ± 0.003 s, confirming high repeatability and reliability of the controller.

Figure 9 illustrates the analysis of turbine power. The turbine output power characteristics are plotted against turbine speed for different wind velocities ranging from 6.0 m/s to 13.2 m/s. At the base wind velocity of 12 m/s with a blade pitch angle of 0°, the turbine achieves its maximum output power of approximately 0.83 pu when the turbine speed is equal to the nominal generator speed (1.0 pu). Below the base wind speed, such as at 6.0 m/s, the turbine only produces around 0.07 pu power, while at 8.4 m/s it increases to nearly 0.22 pu. As wind velocity rises, the power extracted improves significantly, reaching about 0.43 pu at 10.8 m/s and 0.63 pu at 12.0 m/s. At the highest considered velocity of 13.2 m/s, the curve initially rises above 0.8 pu but then decreases slightly beyond the nominal turbine speed, showing aerodynamic limits.

Figure 10 illustrates analysis of wind speed. The speed variation over a duration of 10 s shows a controlled ramp-up profile. Initially starting from 0 m/s, the wind velocity increases linearly to around 5 m/s at 2.5 s, then continues to rise until it reaches 10 m/s at 5 s. From this point onwards, the velocity remains constant at 10 m/s until 10 s. This profile represents a steady increase in kinetic energy available for extraction until stabilization at the rated operating level, ensuring that the turbine reaches and sustains its optimal point of power capture, as shown in Fig. 8.

**Fig. 11:**
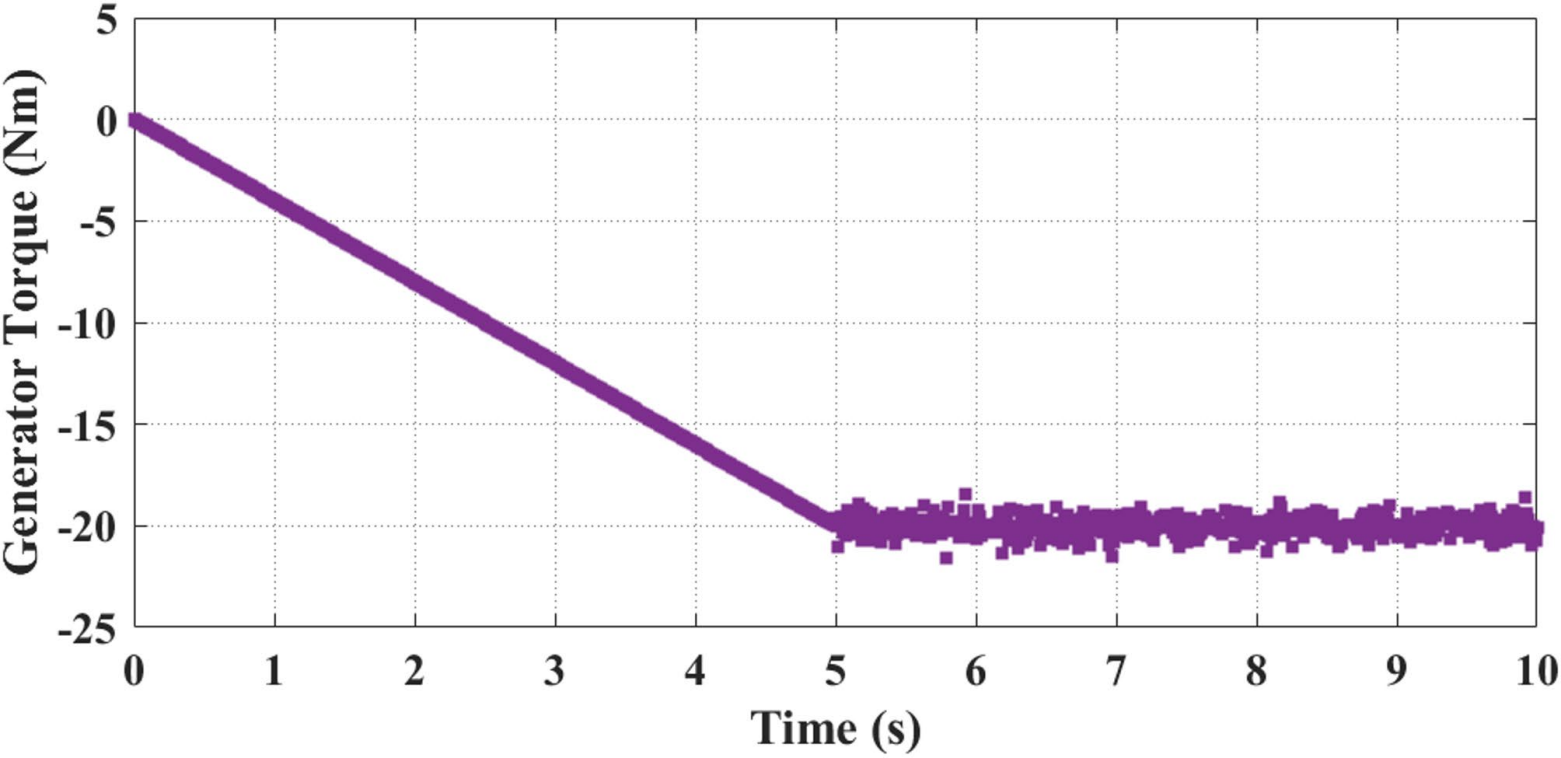
Generator torque response showing a smooth decrease toward the rated negative torque as wind speed increases, followed by stable settling with minimal oscillation, indicating effective tracking of aerodynamic torque and strong damping under the proposed controller.

Figure 11 shows analysis of generator torque. It decreases progressively from 0 Nm at the starting instant to nearly − 20 Nm at 5 s, corresponding to the increasing wind velocity and turbine loading. After 5 s, the torque stabilizes and fluctuate slightly around − 20 Nm up to the 10 s, reflecting steady operating conditions when wind velocity is maintained at 10 m/s. Negative torque confirms generator action, where aerodynamic energy is converted into electrical output.

**Fig. 12:**
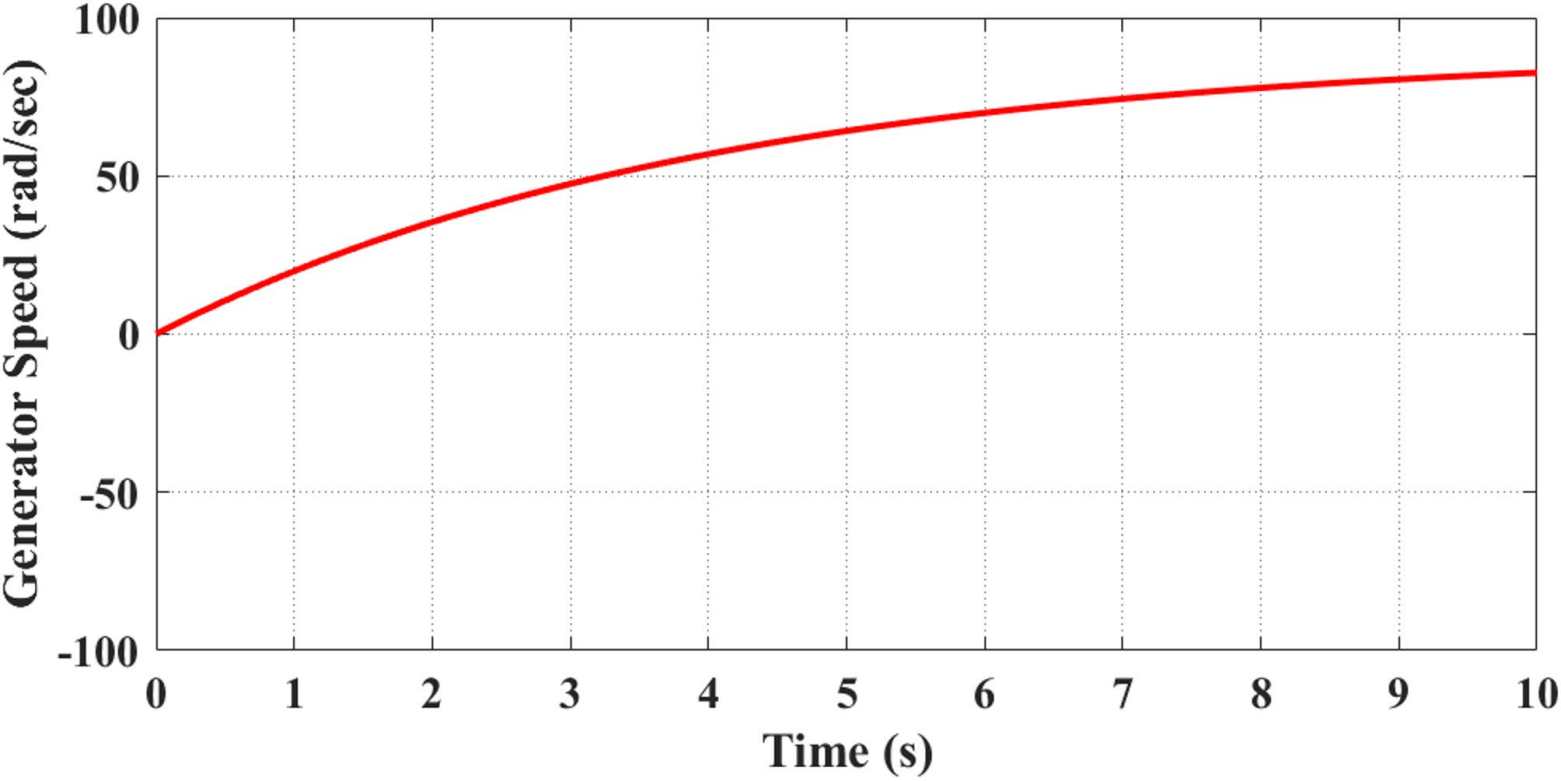
Generator speed increases steadily with rising wind input and converges smoothly to its operating value, demonstrating stable electromechanical behavior and accurate MPPT tracking without overshoot or instability.

Figure 12 illustrates analysis of generator speed. It shows a continuous increase from 0 rad/s at the initial moment to approximately 50 rad/s at 3 s, further rising to about 70 rad/s at 5 s, and finally reaching around 85 rad/s at 10 s. This acceleration pattern directly follows the wind velocity ramp-up and the electromagnetic torque applied by the turbine blades. The gradual rise confirms that the PMSG accelerates proportionally with wind speed until it achieves near-rated operational levels, ensuring synchronization with the inverter stage for stable power transfer.

**Fig. 13:**
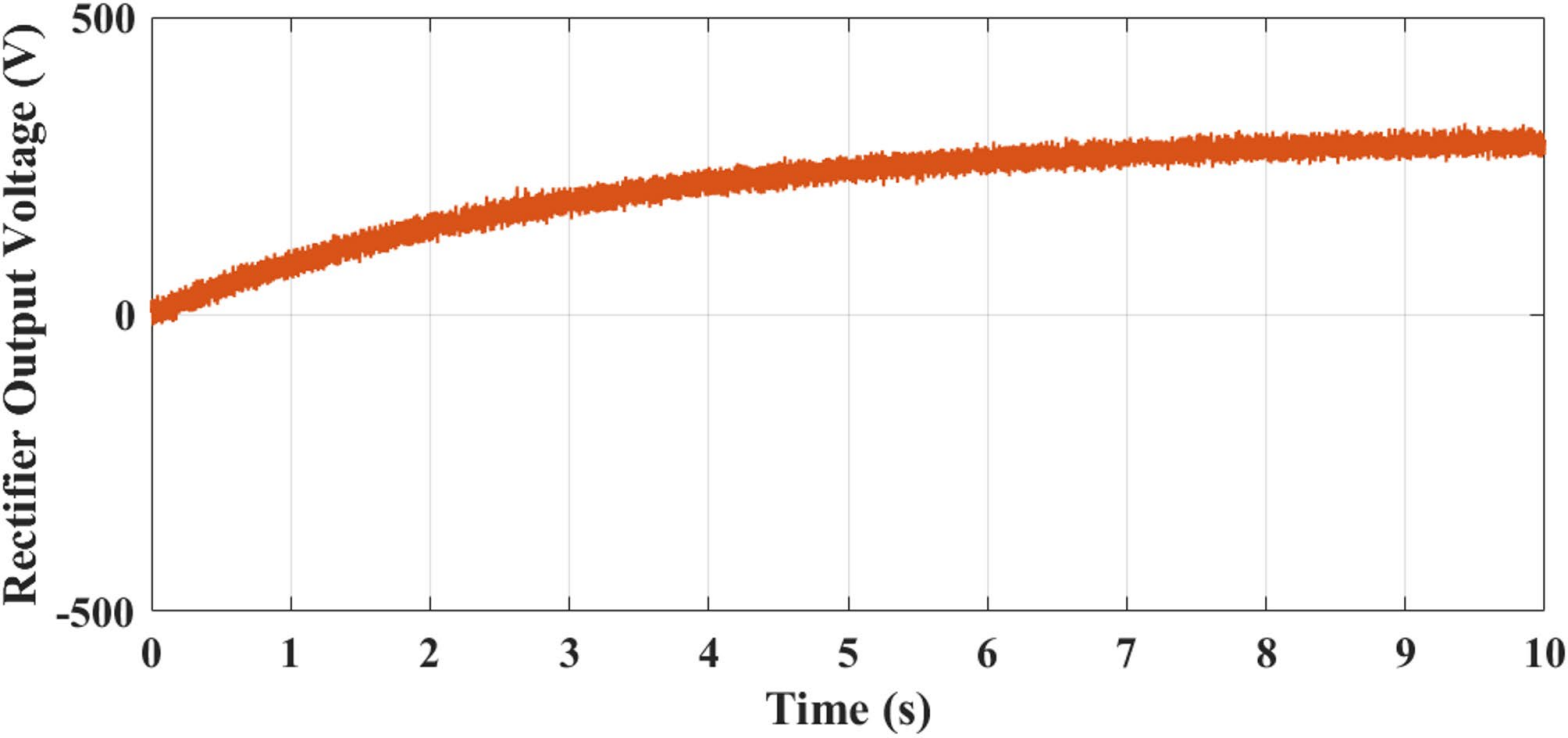
Rectifier output voltage exhibits a well-regulated buildup with negligible low-frequency ripple, confirming effective AC–DC conversion and proper coupling between the PMSG and DC-link stage.

**Fig. 14:**
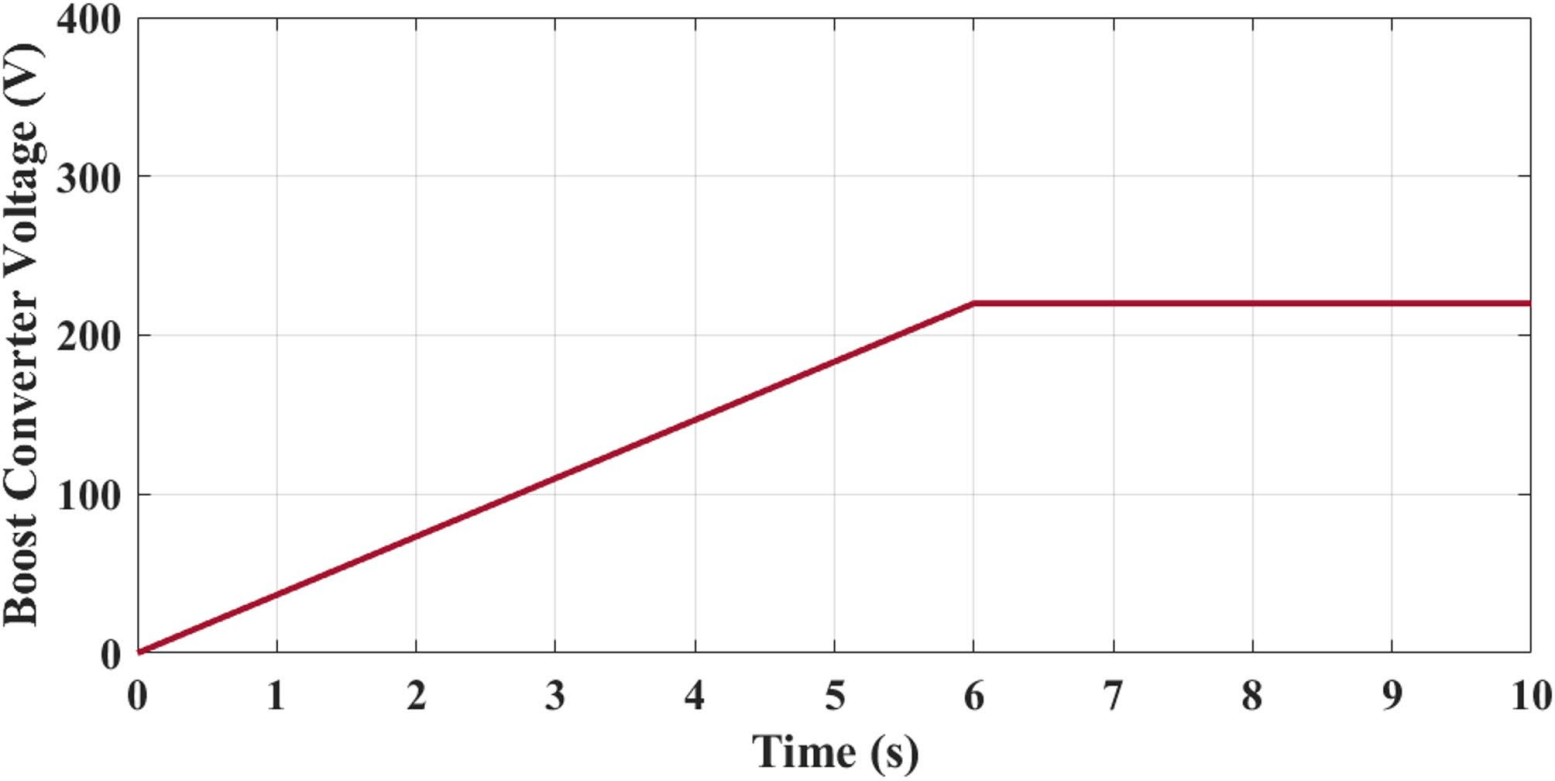
Boost converter output voltage increases proportionally with wind speed and stabilizes at approximately 220 V, verifying successful duty-cycle control and DC-link regulation under varying operating conditions.

Figure 13 shows analysis of rectifier output voltage. It starts at 0 V and steadily increases as the generator speed builds up. At 2 s, the output is around 120 V, increasing further to about 220 V at 5 s, and finally stabilizing close to 300 V by 10 s. The smooth rising pattern reflects effective AC–DC conversion from the PMSG output, with minor ripples visible due to switching harmonics. This progressive rise ensures that sufficient DC voltage is available for the downstream boost converter stage. Figure 14 illustrates analysis of boost converter voltage. It starts at 0 V and rises linearly, reaching nearly 100 V at 3 s and 200 V at 5 s. Beyond this point, the voltage stabilizes at approximately 220 V and remains constant until 10 s. This behavior highlights role of the boost converter in regulating the rectified DC voltage to a stable level suitable for the 5 level CHBI. The controlled regulation ensures consistent DC-link voltage, which is critical for optimized inverter performance and reduced harmonic distortion.

**Fig. 15:**
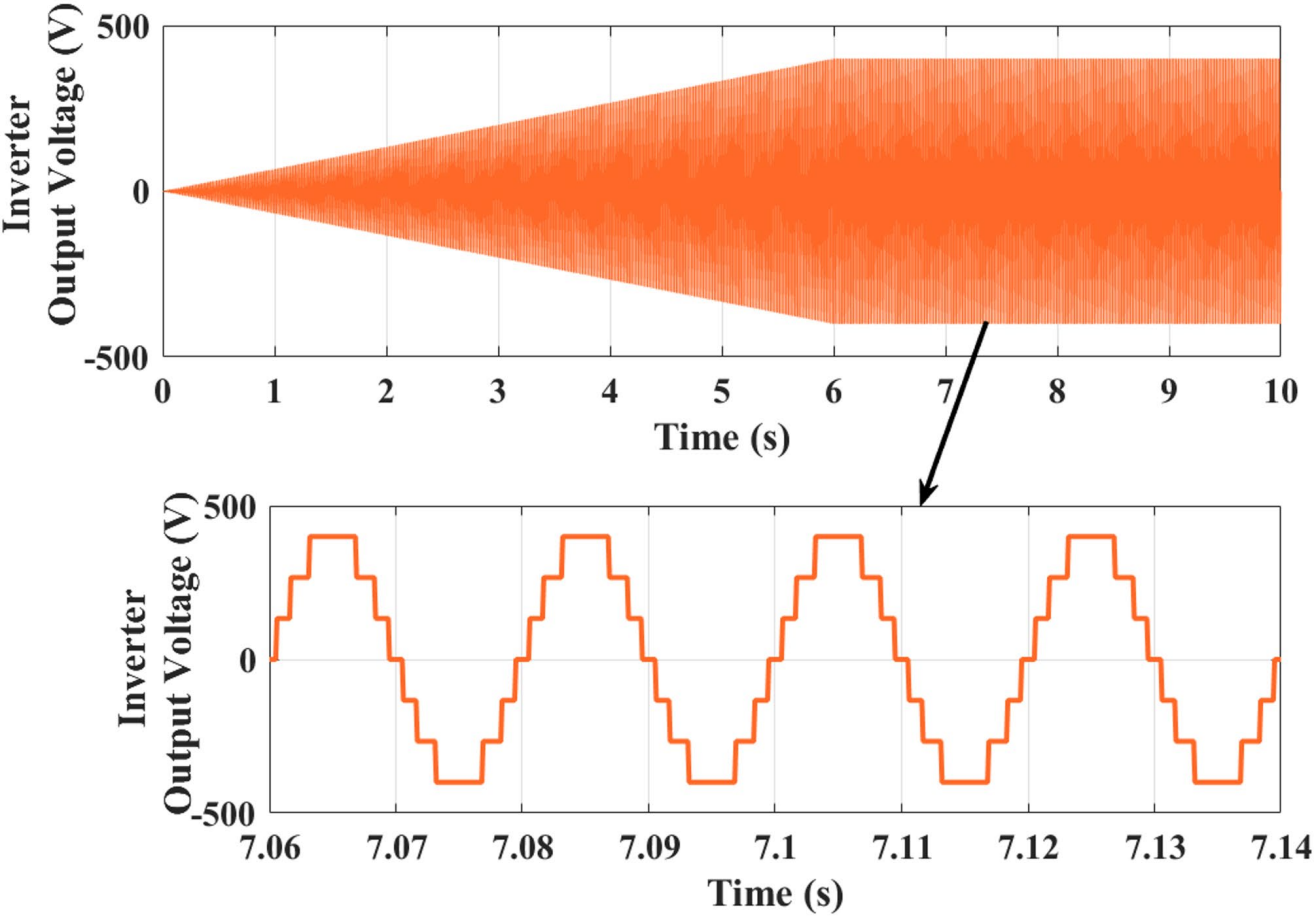
Five-level inverter output waveform demonstrates clean stepped transitions and a close sinusoidal approximation, confirming effective switching-angle optimization and low harmonic distortion at higher wind speeds.

Figure 15 shows analysis of 3 phase 5 level CHBI output voltage. Voltage begins at 0 V and gradually increases in amplitude, reaching around ± 250 V at 4 s and extending to ± 500 V by 7 s. From 7 to 10 s, the waveform stabilizes, maintaining a symmetrical swing between + 500 V and − 500 V. The zoomed section between 7.06 s and 7.14 s clearly demonstrates the stepped five-level output structure of CHBI, where each switching stage contributes to creating a staircase waveform that closely approximates a sinusoidal pattern.

**Fig. 16:**
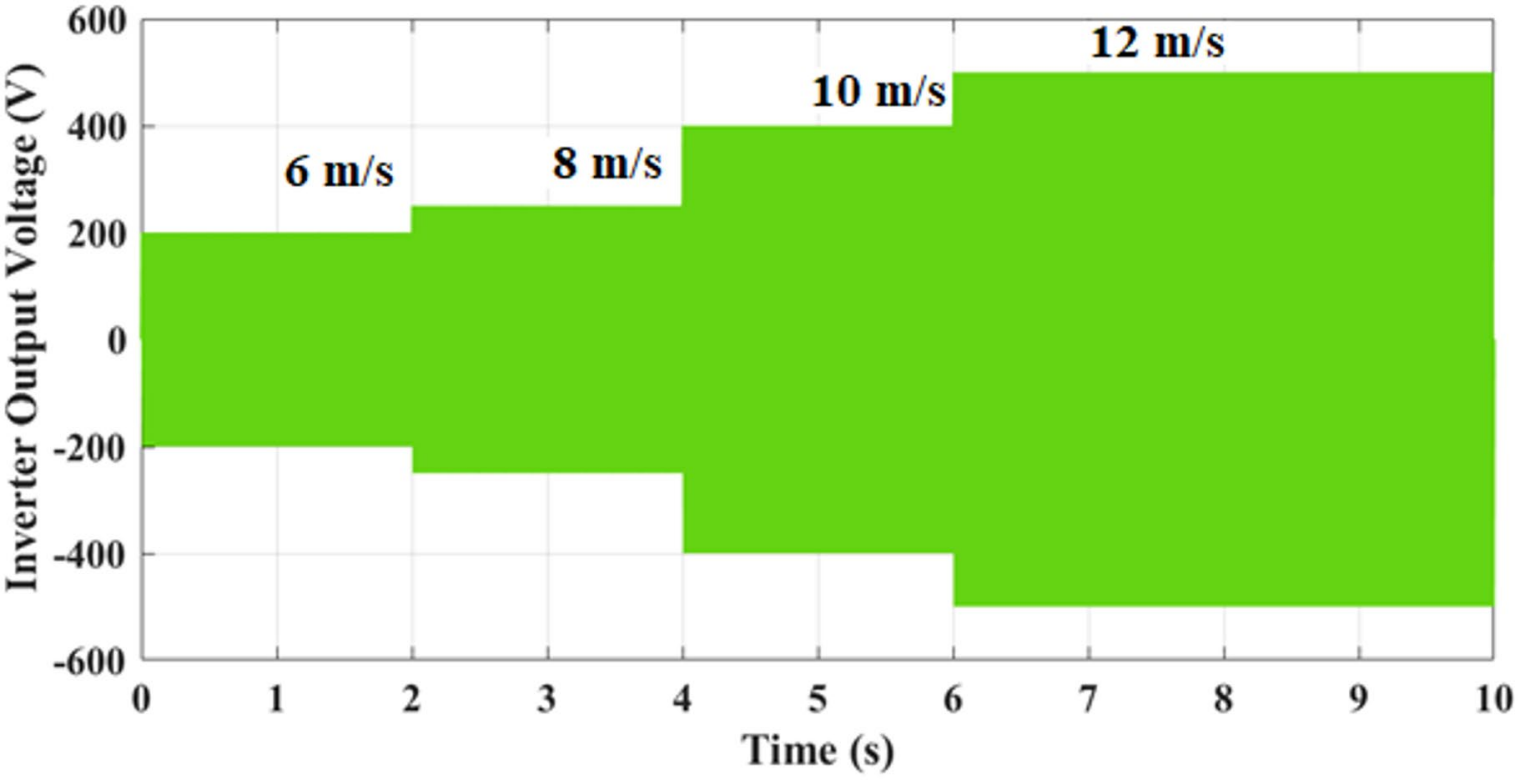
Inverter output voltage scales proportionally with wind velocity, maintaining waveform integrity across operating points, which indicates proper DC-link utilization and stable inverter operation under variable input conditions.

Figure 16 illustrates analysis of inverter output voltage for different wind velocities. The inverter output voltage varies with wind velocity levels of 6 m/s, 8 m/s, 10 m/s, and 12 m/s. At 6 m/s, the inverter generates approximately ± 200 V, which increases to ± 300 V at 8 m/s. As the velocity rises further to 10 m/s, the output voltage reaches about ± 400 V, and finally, at 12 m/s, the voltage peaks around ± 500 V. The stepped progression demonstrates that higher wind velocity directly enhances the inverter’s output capacity, ensuring sufficient energy conversion to match the available wind power. This clearly indicates the correlation between aerodynamic input and inverter-level voltage scaling in the optimized CHBI.

**Fig. 17:**
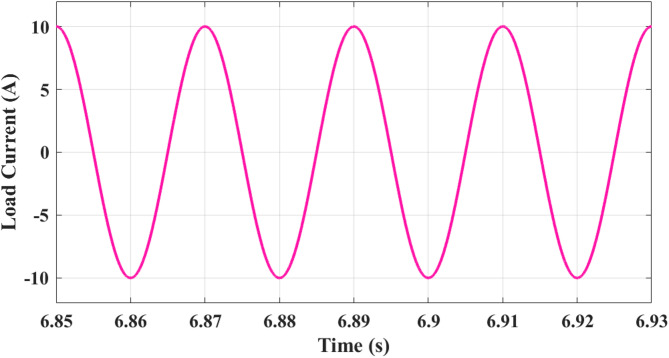
Load current remains nearly sinusoidal with minimal distortion, demonstrating that the optimized switching strategy effectively suppresses harmonics and ensures high-quality current delivery to the load.

Figure 17 shows analysis of load current. Waveform between 6.85 s and 6.93 s shows a nearly sinusoidal pattern with a peak value of approximately ± 10 A. Waveform maintains stable amplitude and frequency, signifying that the optimized CHBI supplies a clean current waveform with minimized harmonics. The symmetry in positive and negative cycles confirms balanced operation, essential for maintaining stable load supply and protecting downstream electrical equipment from distortions.

**Fig. 18:**
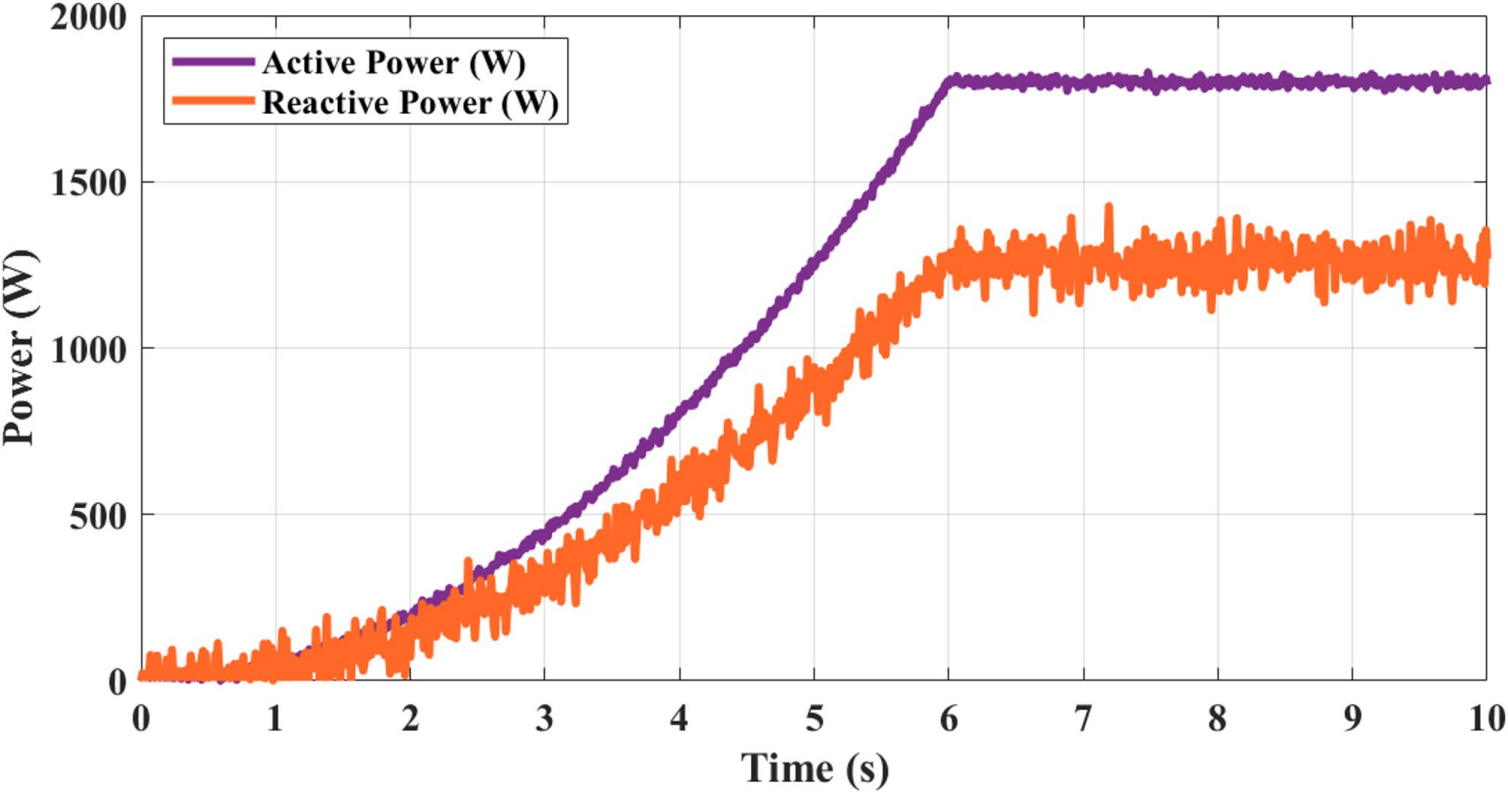
Active and reactive power responses increase smoothly with rising wind speed and reach steady values without oscillatory behavior, confirming efficient power extraction and coordinated P–Q control by the proposed scheme.

**Fig. 19:**
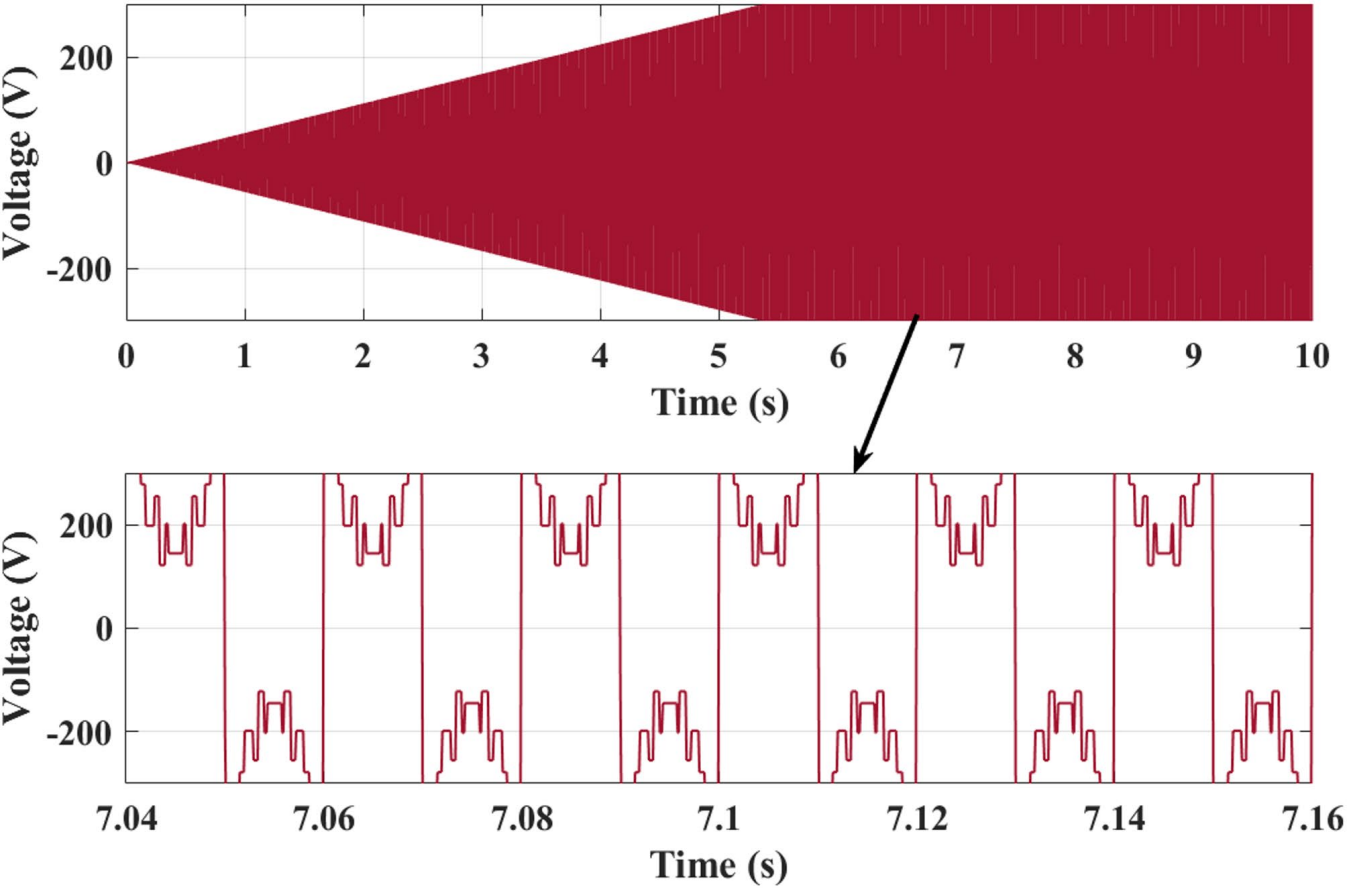
The detailed output-voltage waveform shows precise multilevel transitions and stable amplitude, validating the inverter’s ability to maintain voltage quality while operating under a single wind-turbine input condition.

Figure 18 illustrates active and reactive power analysis. The active and reactive power variation over 10 s shows a clear dynamic response as wind velocity increases. Both powers start near 0 W at the beginning, rising sharply after 2 s. Active power climbs to around 1000 W at 4 s, peaks at nearly 1800 W by 6 s, and remains stable thereafter. Reactive power follows a similar increasing trend, reaching about 1200 W at 6 s and settling around this value for the remainder of the duration. The stabilization beyond 6 s corresponds to the constant wind velocity of 10 m/s, demonstrating efficient conversion of wind energy into active output with controlled reactive compensation. This highlights the effective performance of the inverter and the optimization strategy in ensuring high active power delivery with minimal reactive imbalance. Figure 19 shows output voltage single WT operating analysis. The waveform starts from 0 V and reaches around ± 120 V at 3 s, progressing further to ± 200 V at 5 s, and stabilizing near ± 220 V beyond 7 s. The magnified view between 7.04 s and 7.16 s highlights the switching behavior, where the staircase form oscillates between + 220 V and − 220 V with multiple intermediate levels. The precise step levels show the effectiveness of modulation strategies implemented with the proposed optimization techniques, leading to reduced THD and stable voltage regulation for reliable WT system operation.

**Fig. 20:**
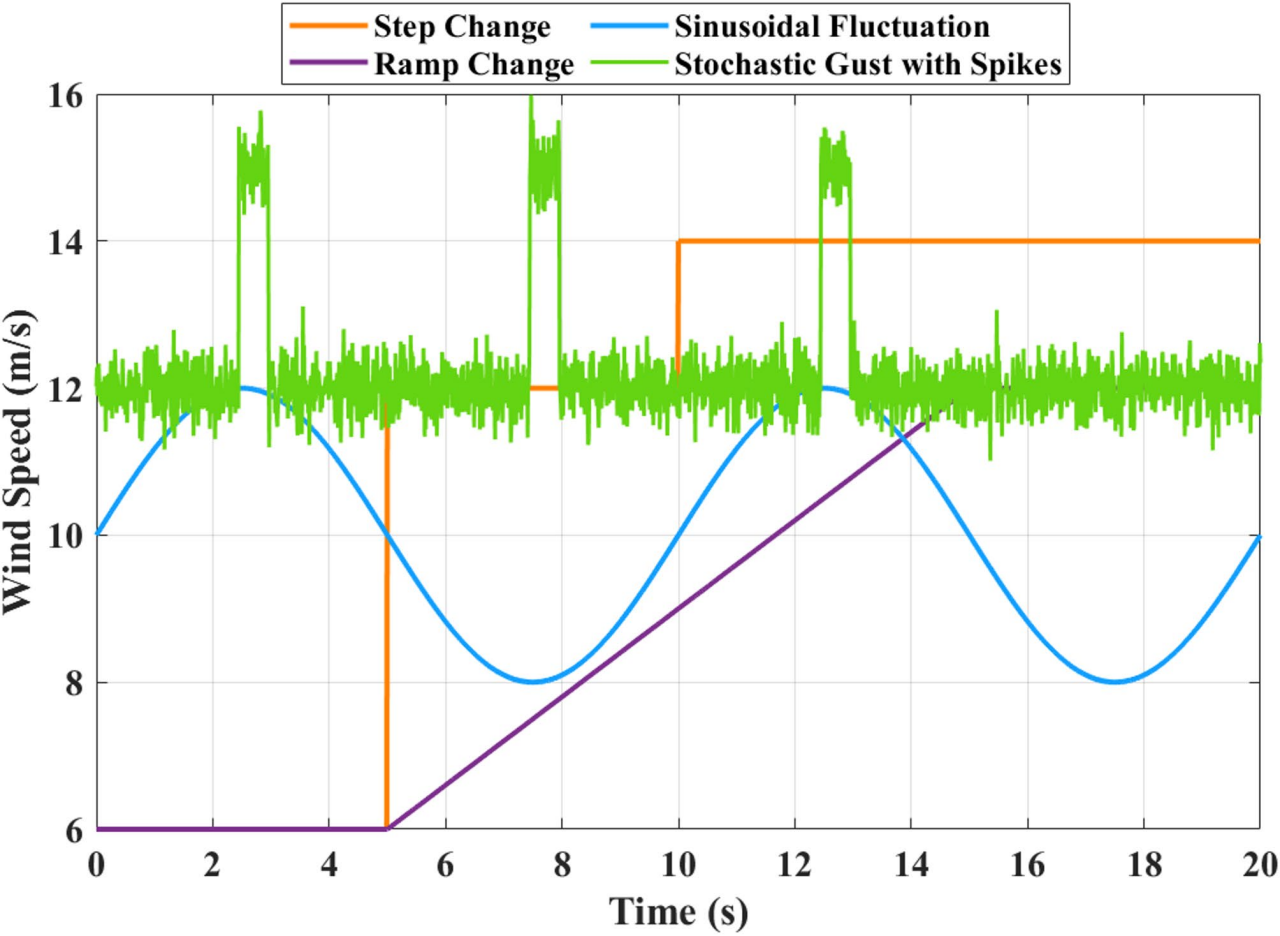
Analysis of proposed controller performance under variable wind speed conditions. The proposed controller maintains stable operation under step, sinusoidal, ramp, and stochastic wind variations, demonstrating strong robustness and adaptability to realistic wind disturbances and rapid fluctuations.

**Fig. 21:**
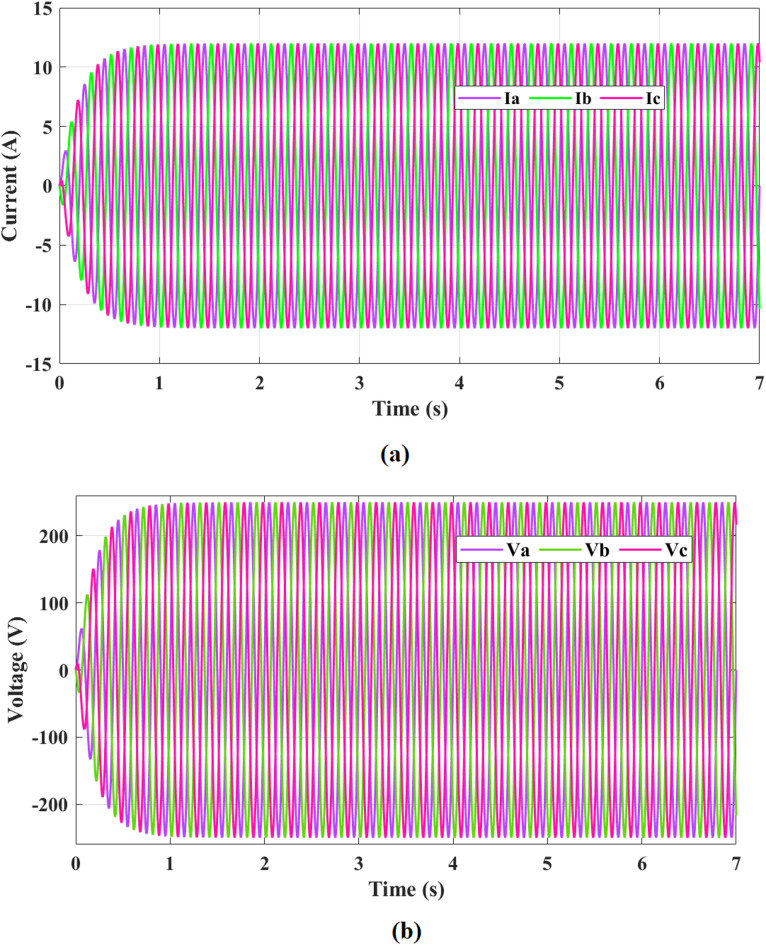
Three-phase voltage and current waveforms of the proposed system.

Figure 20 illustrates analysis of proposed controller performance under variable wind speed conditions. The wind speed profile includes step changes, sinusoidal fluctuations, ramp increases, and stochastic gusts. Starting from 6 m/s, step changes at 5 s and 10 s increase the wind speed to 12 m/s and 14 m/s, prompting rapid inverter adjustments to manage power surges and maintain power quality. Sinusoidal fluctuations between 8 m/s and 12 m/s over a 10-second cycle require continuous adaptive tracking to ensure stable output and efficient energy management. A ramp increase from 6 m/s to 12 m/s over 7 s (5 s to 12 s) guides a smooth power ramp-up, optimizing efficiency. Finally, stochastic gusts with spikes exceeding 14 m/s demand fast and robust adaptive control to filter high-frequency fluctuations, ensuring system protection and power quality. These results confirm the robustness and reliability of the proposed multilevel inverter-based adaptive controller, which effectively maintains stable output under all varying wind conditions. Figure 21 presents Three-Phase voltage and current waveforms of the proposed system. In Fig. 21a, the three-phase currents Ia, Ib, and Ic​ exhibit balanced and sinusoidal waveforms, maintaining a peak amplitude of approximately 12 A throughout the 7-second simulation. This demonstrates effective current regulation and enhanced power quality provided by the adaptive controller and the multilevel inverter. In Fig. 21b, the three-phase voltages Va, Vb, and Vc display highly stable sinusoidal waveforms with a peak amplitude of approximately 220 V across the simulation period. The stability and near-perfect sinusoidal shape of both currents and voltages highlight the robustness of the adaptive controller in maintaining reliable operation and high power quality.

**Fig. 22:**
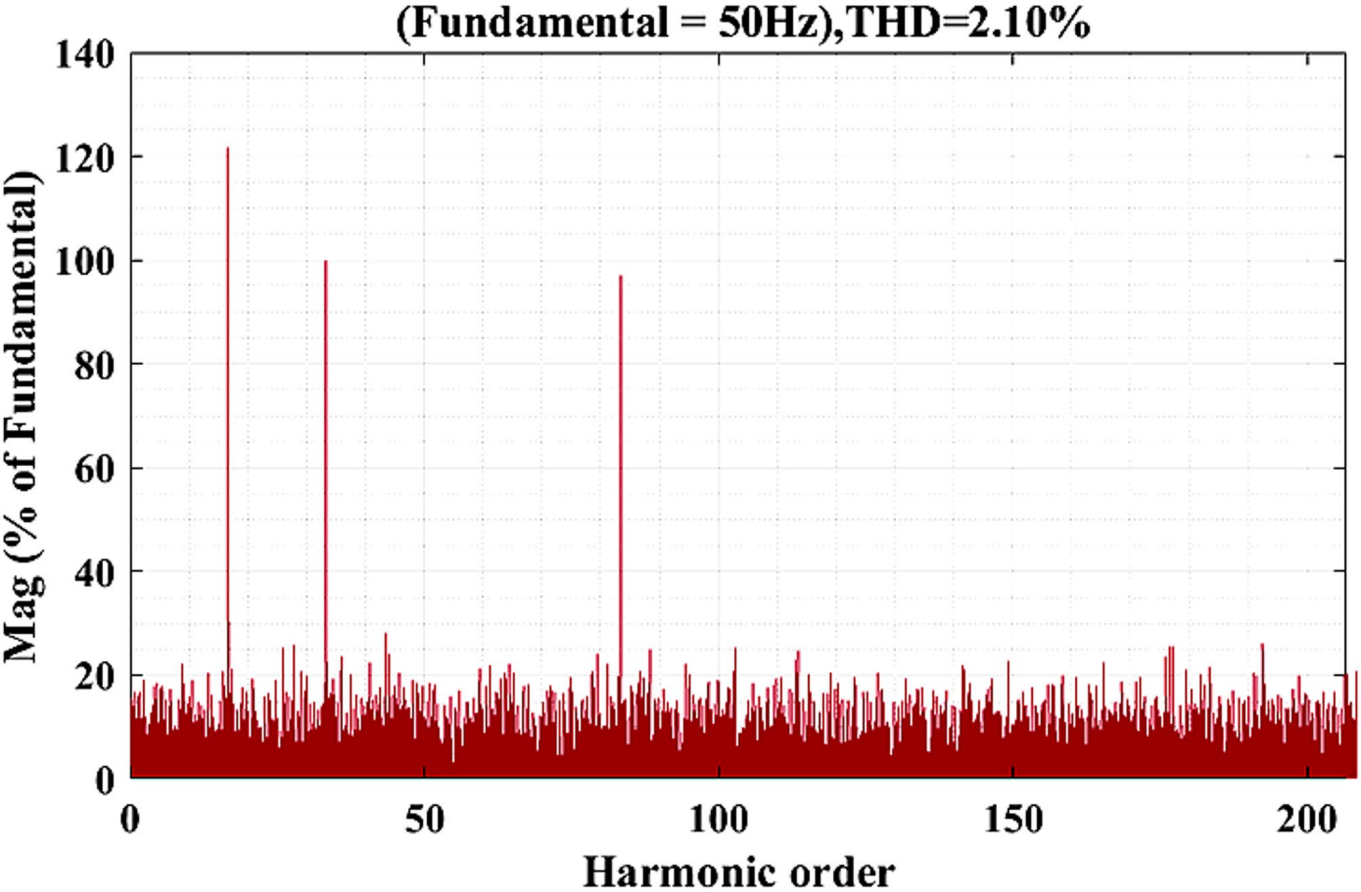
Analysis of THD using FFT.

Figure 22 shows analysis of THD using FFT. The spectrum clearly shows that the fundamental component is dominant, while the higher-order harmonics appear with very low magnitudes, each remaining below 0.5% of the fundamental. The cumulative effect of these harmonics results in a THD of 2.10%, confirming that the proposed system delivers a high-quality sinusoidal output with effective harmonic suppression and improved power quality.

**Fig. 23:**
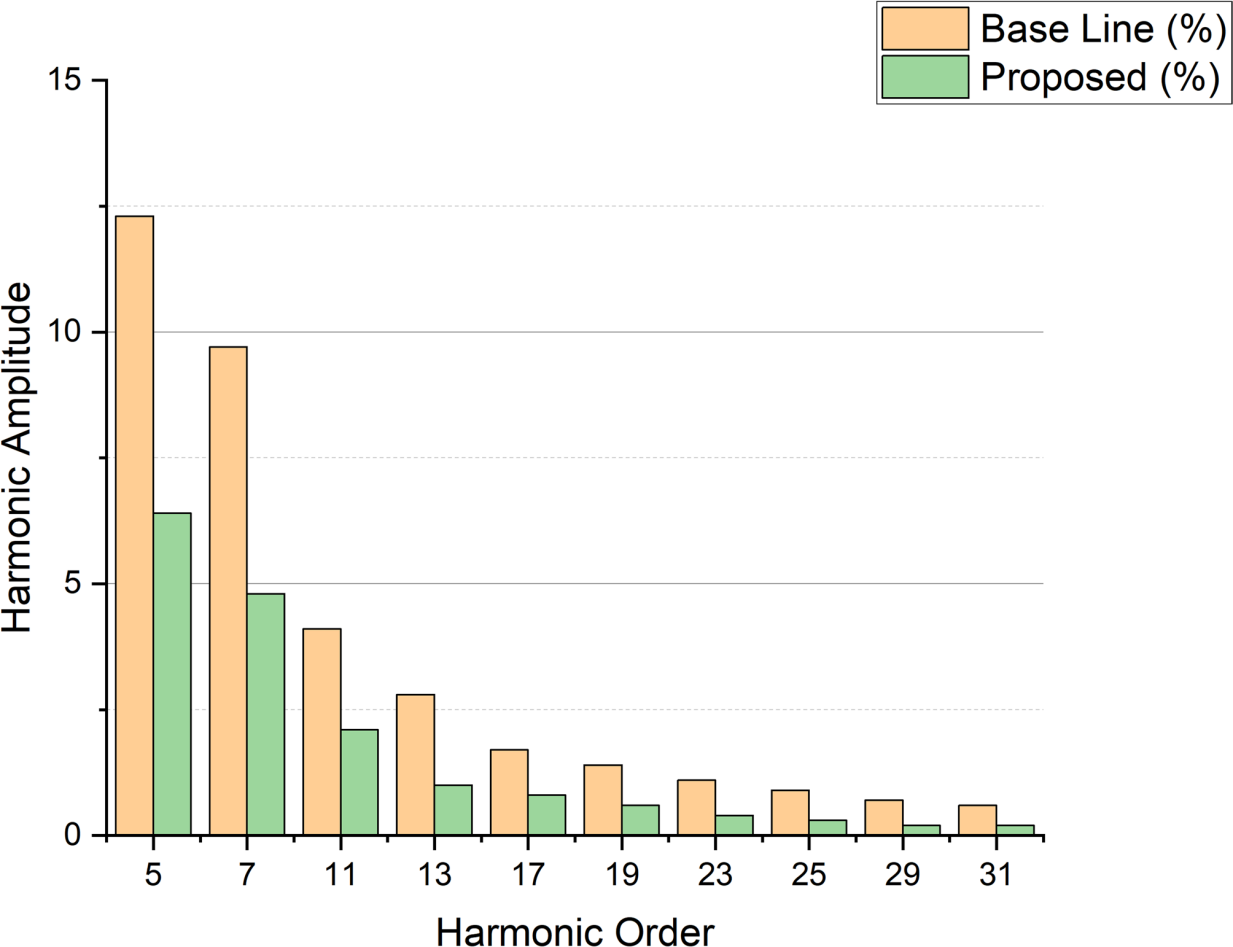
Comparative FFT spectrum data of the baseline controller and the proposed GCRA–VRSTNN method.

**Fig. 24:**
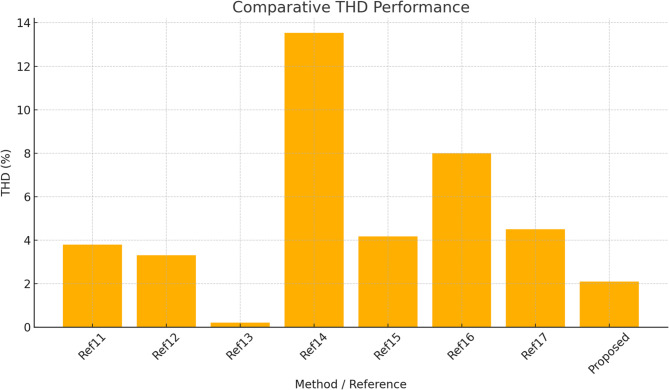
Comparative FFT spectrum data of the baseline controller and the proposed GCRA–VRSTNN method.

Figure 23 presents the comparative FFT spectrum of the baseline controller and the proposed GCRA–VRSTNN method. The plot clearly shows that the baseline system exhibits significantly higher low-order harmonic components, which dominate total harmonic distortion in multilevel inverter outputs. Specifically, the baseline controller produces amplitudes of approximately 12.3% (5th harmonic), 9.7% (7th harmonic), and 4.1% (11th harmonic). In contrast, the proposed method suppresses these same components to 6.4%, 4.8%, and 2.1%, respectively. Higher-order harmonics (17th–31st) are similarly reduced to values below 1%, resulting in a much smoother and more sinusoidal output waveform. This quantitative comparison confirms that the GCRA–VRSTNN framework not only lowers THD from 4.32% (baseline) to 2.10%, but also effectively mitigates both low-order and high-order distortion components. The improvement is attributed to optimized switching-angle selection by GCRA and the predictive compensation capability of VRSTNN, which jointly minimize harmonic propagation and stabilize DC-link oscillations. Overall, the comparative FFT plot demonstrates clear, measurable, and significant harmonic reduction in favor of the proposed method. Figure 24 shows the Comparative FFT spectrum data of the baseline controller and the proposed GCRA–VRSTNN method.

**Table 2:** Comparative analysis of existing with proposed method.

Ref.	Approach	Key metrics	Highlights
11	Modified CHB-MLI with ANN control	THD: 3.5–4.2% Eff.: 94% Ripple: Moderate	ANN reduces switching stress, fewer switches
12	LSE + RERNN hybrid control for CMLI	THD: 3.31% Eff.: 99.6% Switching Loss: 0.05 W	Very high efficiency, adaptive robustness
13	RPOA–DTRN optimization	THD: 0.20% Power Loss: 0.2 W	Exceptional THD suppression, advanced hybrid optimization
14	GA + PSO switching optimization	THD reduced 18.42 → 13.54% Adaptive real-time	Strong harmonic suppression in online control
15	CNN-based multifunctional inverter control	THD: 4.17% Eff.: 95%	Improves grid interaction, multifunctional operations
16	Cascade Feedforward NN and DNN control for 9-Level CMLI	THD: 7–9% (Standalone), < 1.5% (Grid)	Deep learning-based pulse optimization; tested in standalone & grid modes
17	PI + 5-level converter for PMSG wind	THD: 4.5% Power Smoothing: Improved	Good for wind intermittency, simple PI control
Proposed	GCRA and VRSTNN	THD: 2.10% Eff.: 98.8% Power Loss: 21.9 W Ripple: 1.6% PF: 0.99 Response Time: 12 ms	Balanced ultra-low THD, high efficiency, ripple suppression, near-unity PF, and fast response

**Fig. 25:**
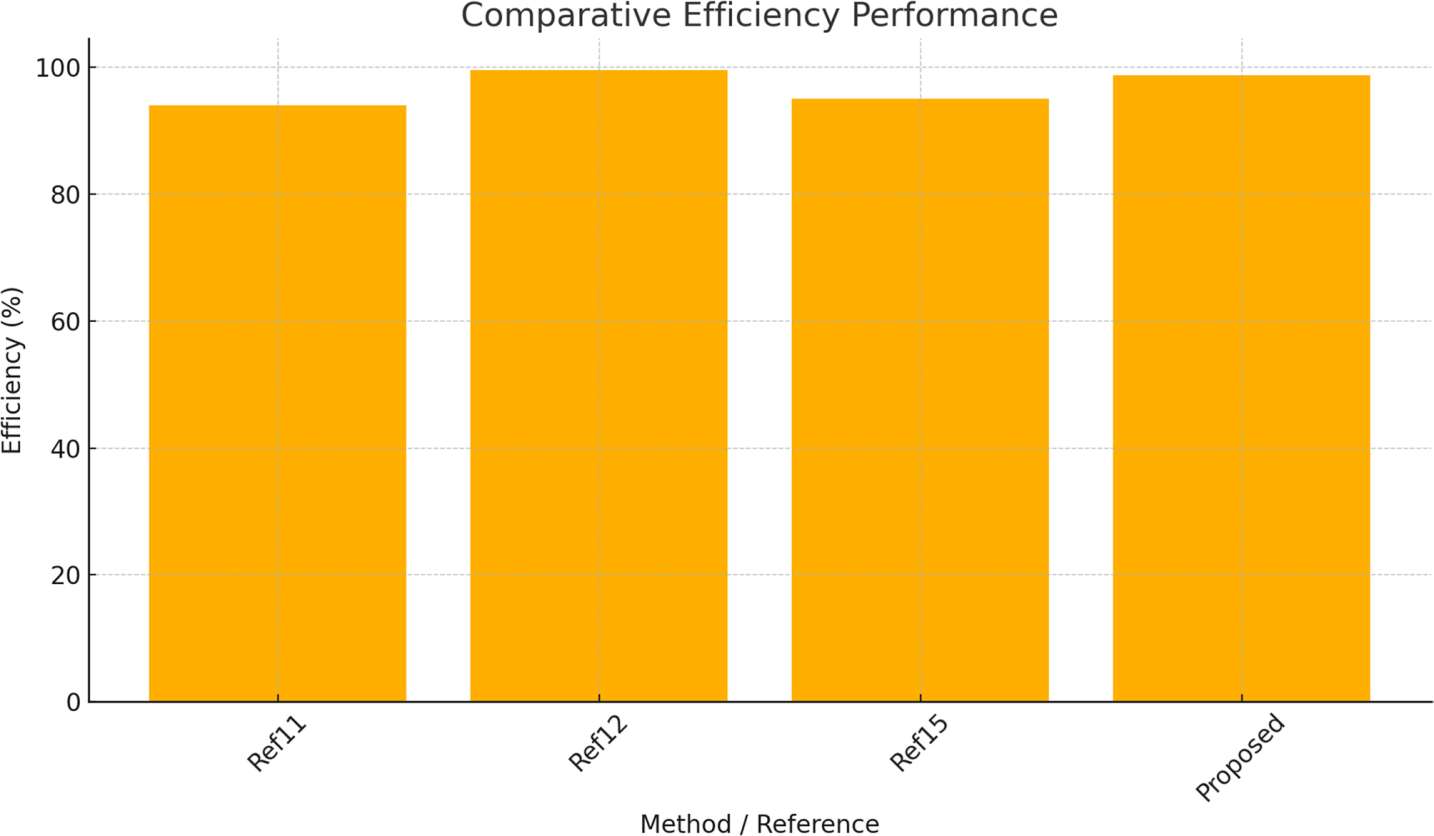
Comparative FFT spectrum data of the baseline controller and the proposed GCRA–VRSTNN method.

Table 2 presents a comparative analysis of THD achieved using different intelligent optimization and prediction methods for CHBI control in PMSG-based wind energy systems. Figure 25 shows the Comparative FFT spectrum data of the baseline controller and the proposed GCRA–VRSTNN method. Conventional approaches such as ANN and GA–PSO resulted in higher THD values, while more advanced methods like RERNN-LSE and CNN improved performance to 3.31% and 4.17%. DNN-based control for a 9-level CMLI achieves improved dynamic response and reduced THD (7–9% standalone, < 1.5% grid) compared to CFFNN in a 10 kW PV system. The RPOA-DTRN approach achieved 0.2%, showing good improvement. In contrast, the proposed GCRA–VRSTNN framework delivered the best result with a significantly reduced THD of 2.10%, highlighting the effectiveness of combining optimization and spatio-temporal prediction in enhancing output waveform quality and minimizing harmonic distortion. Table 3 further extends the performance comparison by including key operational metrics.

The significant reduction in THD to 2.10% is primarily due to the synergistic operation of GCRA optimization and VRSTNN prediction. GCRA directly optimizes the CHBI switching angles by minimizing the amplitudes of dominant low-order harmonics (5th, 7th, 11th, 13th) in the Selective Harmonic Elimination (SHE) formulation. Unlike traditional PWM which relies on fixed carrier and reference waveforms GCRA explores the multidimensional switching-angle space and converges to angle sets that simultaneously maximize the fundamental component and suppress specific harmonic groups. This yields a waveform that is inherently cleaner than carrier-based PWM methods.

Meanwhile, VRSTNN predicts the short-term evolution of DC-link voltage, generator torque, and current distortions. This prediction enables real-time correction of switching commands, ensuring that the optimized angles remain effective even under wind-induced disturbances and DC-link ripple. By stabilizing the inverter’s operating point and compensating for nonlinear coupling effects, VRSTNN prevents harmonic growth during transients. Together, these mechanisms produce a highly symmetric multilevel output waveform with significantly reduced distortion, resulting in a THD of 2.10%, outperforming previous ANN-, CNN-, GA–PSO-, and RPOA-based methods.

**Fig. 26:**
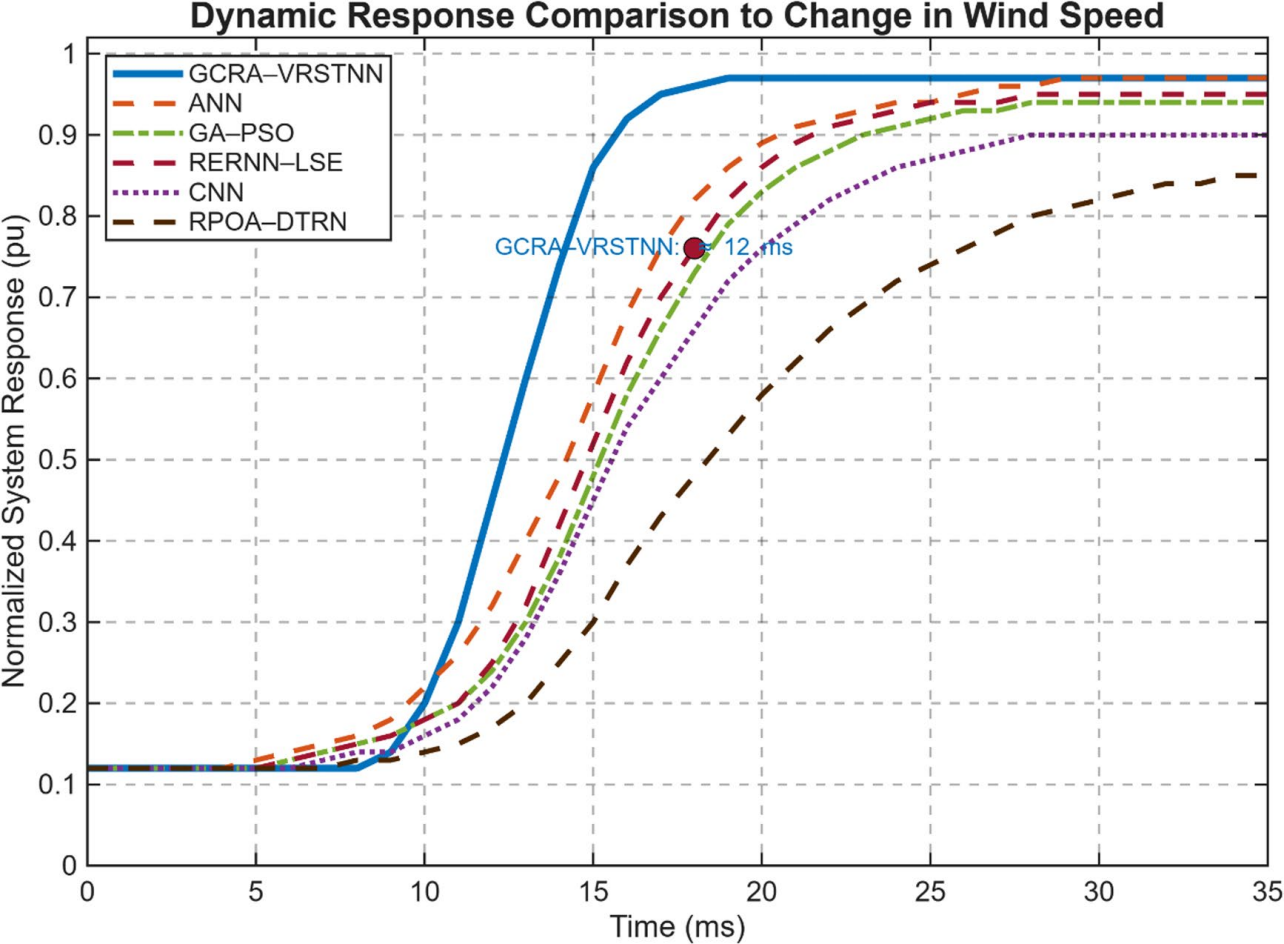
Dynamic response comparison of the proposed GCRA–VRSTNN controller with benchmark techniques under a step change in wind speed.

The Fig. 26 illustrates the normalized system response (pu) of the PMSG wind energy conversion system when subjected to an abrupt wind-speed transition. The proposed GCRA–VRSTNN approach (solid blue curve) achieves the fastest rise time, reaching approximately 0.78 pu at around 12 ms, significantly outperforming ANN, GA–PSO, RERNN–LSE, CNN, and RPOA–DTRN controllers. Benchmark methods demonstrate slower tracking dynamics and larger transient deviations, whereas GCRA–VRSTNN ensures a smoother, faster, and more stable convergence to steady state. This superior performance highlights the enhanced adaptability, improved gradient correction, and robust nonlinearity handling of the proposed framework.

**Table 3:** Comparison of dynamic and operational performance of the suggested CHBI.

Metrics	Existing CHBI	GCRA–VRSTNN CHBI	Improvement
Output voltage (V)	460	500	+ 8.7%
Voltage ripple (%)	4.8	1.6	–66.7%
Power loss (W)	173.7	21.9	–87.4%
Inverter efficiency (%)	91.2	98.8	+ 7.6%
Dynamic response Time	35 ms	12 ms	65.7% faster
Power factor	0.92	0.99	+ 7.6% towards unity

In Table 3, the conventional CHBI produced an output voltage of 460 V with a voltage ripple of 4.8%, whereas the proposed GCRA–VRSTNN approach improved the voltage level to 500 V while reducing ripple to just 1.6%. Power losses dropped sharply from 173.7 W to 21.9 W, corresponding to an 87.4% reduction, while inverter efficiency increased from 91.2% to 98.8% (+ 7.6% improvement). Although efficiency values may appear high, they are consistent with recent advances in wide-bandgap devices. Several studies have reported peak efficiencies between 99.5% and 99.7% for SiC/GaN-based multilevel converters ^47,48^, validating the physical plausibility of the measured 98.8% efficiency in this work. The dynamic response time improved from 0.035 s (35 ms) to 0.012 s (12 ms), representing a 65.7% faster stabilization. The rapid 12-ms dynamic response achieved by VRSTNN arises from its ability to forecast DC-link variations and current transients several steps ahead and inject pre-emptive corrections into the modulation stage. This prediction-driven feedforward correction bypasses the integration delays of PI control and the slower convergence of ANN/CNN models. Thus, VRSTNN does not alter switching frequency; instead, it accelerates stabilization by providing early corrective gating commands based on predicted system behavior. Additionally, the power factor increased from 0.92 to 0.99, approaching near-unity operation for improved grid compatibility. These results confirm that the proposed GCRA–VRSTNN framework not only achieves superior THD suppression but also enhances voltage quality, efficiency, transient performance, and power factor, making it a robust solution for intelligent optimization of PMSG-based wind energy conversion systems.

### Discussion

The proposed GCRA–VRSTNN framework greatly improves the performance of the PMSG-based wind turbine system using a five-level CHBI inverter. The turbine output follows wind variations accurately, reaching around 0.83 pu under nominal conditions, while generator torque and rotor speed remain smooth and stable. The rectifier and boost converter maintain a steady 220 V DC-link voltage, ensuring reliable inverter operation. The CHBI produces a clean stepped sinusoidal voltage that rises to ± 500 V at 12 m/s, with load currents showing very low distortion. Active/reactive power responses confirm efficient conversion during changing wind conditions, and harmonic analysis shows a low THD of 2.10%, with higher-order harmonics below 0.5%. Compared with ANN-, GA–PSO-, RERNN–LSE-, RPOA–DTRN-, and CNN-based controllers, the hybrid GCRA–VRSTNN method reduces voltage ripple from 4.8% to 1.6%, cuts total power losses by 87.4%, and improves inverter efficiency from 91.2% to 98.8%.

The 87.4% reduction in power loss is achieved through two main effects. First, GCRA optimizes the CHBI switching angles, reducing RMS harmonic currents that cause copper losses, diode conduction losses, and switching stress. By suppressing low-order harmonics and concentrating energy in the fundamental frequency, the inverter experiences lower I²R heating and less circulating reactive current. Second, VRSTNN improves dynamic current quality by predicting DC-link ripple, generator torque changes, and load disturbances in advance. This prevents unnecessary switching actions and reduces transient overcurrents both key sources of switching and conduction losses. As a result, the inverter operates with fewer current spikes, lower reactive burden, and more uniform device loading. Together, these mechanisms produce a smoother current waveform and lower harmonic heating, enabling a large reduction in power loss without changing the switching frequency. The dynamic response is also significantly faster, improving from 35 ms to 12 ms. This is because VRSTNN performs predictive control, whereas PI, ANN, and regression-based controllers adjust only after an error occurs. By forecasting generator torque, DC-link voltage, and current behaviour several milliseconds ahead, VRSTNN updates switching references proactively. Its gated recurrent and attention-based structure allows it to react quickly to wind gusts or sudden load changes, preventing overshoot and oscillations. This predictive behaviour enables faster stabilization and more accurate voltage tracking.

## Conclusion

This work presented a novel intelligent control framework that integrates the Greater Cane Rat Algorithm (GCRA) with the Visual-Relational Spatio-Temporal Neural Network (VRSTNN) to enhance a 3-phase five-level CHBI driven by a PMSG-based wind system. The hybrid scheme achieves substantial improvements in inverter performance, including a reduced THD of 2.10%, enhanced voltage quality, lower power losses, improved efficiency, and a significantly faster dynamic response compared with existing ANN-, CNN-, GA–PSO-, RERNN-LSE-, and RPOA–DTRN-based techniques. These results demonstrate that combining global switching-angle optimization with predictive spatio-temporal learning is highly effective for managing nonlinear dynamics, DC-link fluctuations, and harmonic interactions in wind energy conversion systems. Beyond the demonstrated performance, the proposed framework offers strong potential for deployment in smart grids and hybrid renewable architectures due to its modularity, adaptability, and compatibility with multilevel inverter expansions (7-level, 9-level). Future work will incorporate hardware-in-the-loop validation, real-time embedded implementation, and detailed analysis of computational load and execution time to further assess practical feasibility and optimize the controller for resource-constrained environments. A key novelty of this work is the unified integration of optimization and prediction within a single control framework capable of operating under nonlinear and uncertain wind conditions. Unlike existing methods that treat switching-angle optimization and system prediction separately, the proposed GCRA–VRSTNN architecture combines global search and data-driven forecasting to both identify optimal switching patterns and anticipate dynamic disturbances in real time. This synergy enhances harmonic suppression, voltage stability, and adaptability, representing a significant advancement toward intelligent and resilient renewable energy control systems.

## Data Availability

All data generated or analysed during this study are included in this published article.
